# Supplementary material for: Selfish conflict underlies RNA-mediated parent-of-origin effects
Source: Nature. 2024 Mar 6;628(8006):122–9. doi: 10.1038/s41586-024-07155-z (PMC10990930; doi:10.1038/s41586-024-07155-z)
Supplement: Supplementary file 1 — Supplementary Methods, Supplementary Fig. 1, Supplementary Tables 1–7, Supplementary Discussion, Supplementary Notes 1–3 and references. [file 41586_2024_7155_MOESM1_ESM.docx]

# **Supplementary Information**

**Supplementary Methods**

**Annotation of *C. tropicalis* Argonautes**

We annotated Argonaute proteins in *C. tropicalis* (NIC203), reconstructed their phylogenetic relationship, and named them based on homology to *C. elegans* Argonautes (see Methods and Extended Data Fig. 2). Argonautes are a large family of fast evolving proteins that have experienced extensive gene duplication. Therefore, it is recommended to perform functional characterizations of these homologs before concluding orthology. Here we summarize the sequences of all *C. tropicalis* Argonautes used to generate Extended Data Fig. 2.

>Ctr-ERGO-2.2
MSYNNRYNDRRDDRRDDRGQYNDRYDDRRDGNESRQSGSDRSQYGGSQYGGSQHGGSQYGGSQYSQHSGDSRRGGRGDYG
RGGSRGGGRGGHQNGRDDLSHQMGRMNMNSNIYSQHGNPRCPRPVPQVGDLAGSAHQPRMPIPKKDLAHNTQKFAERPDS
WVYDKKAVGTKTTLLTNHALVHLPEQTVQLYCYNIEIFNNKKLVNNREQASPLFHAYVRKFTRDFPNRRDYVFNDVNILW
TSSRLPKSEGRVGDNRSWMMYKYTTRFDFGQTIREDQDKQLLSTLVDAIATARVRSGCPKFTVFKRCMFMIGDGTTDNQI
EAPLSFPVGQGLDARCGISIGVRINLRAGVTACYDISHTIFNRPGYPLIRLLCELINAGPISDEEFEEVWDERLRGAVMT
ERNFNELDRILKKMKLRYSLETAVSIDENGKVIVKKIPRDPKDFKYYQLTATSAQDTTFRDDKGKTWSIAEYFLEVSHIR
LRYPNLPCFVKKPSKQNTRFIAFPMEFVSYVTDAVRYEGNPGDRIKMEMIRQSAYTASQRRALLQHIIEQKPIADVPAAI
DNNDSYMRNHGLSIEKEMLSVKATILPPPTVVYGEGSKFTDVHHIGEWEAITHDPIRKVLEGSVYVRNKDGPQLKKRLLC
SVIHISSPENGKTEISYDPACYHRLMNAIEEAGQPVAWENYEERYAAMHGVLNYCQGADDPYVIMEFFQNIKANIDNKYK
QSEDEVIIPFVLLVFQIKCSTLKGHRDQWDNDYNFIKYLGDSEVGIYTQGILAENLCAIKETPAICKLTRLLVEKILGKV
GTTHRKLEREGDHKSWTKLTDPKSPTLVLGVDVSHPSPRDRLEHDIRKLSVATVVGNIDFDCTEYRASSRIQDAGEENIV
RFREEITARLNAFIQNTGVRPKHIVVYRDGICEGDFQRILYEERINIEQCCVDMDPGYQPTITYIVVSKRHHTRFFLKDN
AEGHESQGFNVLPGTLVEDTVTTKEYYDFYLTSQVGQLGVAHPTHYYVLHDTWKPMVSFWPTVTHALTYMFCRATSTVSL
PAPVLYAHLAAKRAKECLDGAWTVARFRNMQLSVDSFHDVNKLTQAININAQLDGMTFV

>Ctr-HRDE-1
MSGPFDQIYDNMTTTSKSSTPASSSTPMGRIPKRDNTSSKIQEPSSKKNAPAFVMPNSTTPVGRDSFGNCGNMDIQLNMF
SLDLTRMPERIDRLHVDLFIVGSNGKEFNLNLGNVAVSGDVNNHNRRLATLIMFRKFHEIVRHMFSGKSYHYLAYDSAAA
CYVPSGVYTAGEQQAKLTQDNFSEDEWEIVSKICRRKDDAFIIKVKPAGVVYSQGEAAMDPSNHLELTRVVEIVTSQKLN
TTDFYQFGNATFPLRAPATAEPDATTEIRPGFAKVARIVEGRGGKIEMLMTIDTKSSPFFKECHVLKFVTNKYAESRGFG
GGGGRGGFGGRGRGGGGRGGYGGRADSRDSRGGYYRDDSRDSRGSYGGRSDSRDSRGGYRGDRRDSYSRDDRRDYRDDSR
GRSDPYESRHGGDSARQFDPREVEEAQKALQDNRNLLNSIEFGLKGLYVIATHLKGTNSLIRITAVKENNAENTFFEKKD
DKGVQQISVADYFRDTYNIKLKFPRLPLVVSKRMKYECFYPMEVLQIVPGQRMKIQKMSPNVQSAMTGRNAAMPRQHVEM
VQNILRDSLKLDRNQYMEAFGIELTSTKPINLRAKLLPPAQIVFKNAVYMPTMSSPAFRTQAKFIDPARIRRVAVVSFDR
ALDDRTLEQFCDTLYHCCMDNGIKVESASNWSTREIASADNAAIKEEMERWMRKDVDILVGIHRDKKPDVHDVLKFYEES
VGMQTIQLCRQTVDKMMSGQGGRQTTDNVMRKFNLKCGGTNFHLDIPSSVNGKSICANTETLKRKLFEGVQFIGFEISHG
AARTLYDRSRGQMDGEPSIVGISYSLTNSTQLGGFSYMQTSREYKLQKLDEHFPMCVNMYNEHSKKLPSRIVIYRVGAGE
GDFKRCKEEVEEIRATFDKIQAGYKPQLVVIIAQKTSHARVFPLRIEGNRPQEQNVPSGTCIDNVVTSFGYDEFILSSQV
PLIGTVRPCKYTILVNEPGWSKNELIHLTYFRAFGHQVSYQPPSVPDVLYAAENLAKRGKNNYKVYQRYVSIQQIENRLI
EEHADFVNEDMRDKLASAIVDDMSYAMNKMTIRKRNFWA

>Ctr-ERGO-1
MSYNQQRGGGGYNRNQGGNNYQDNYHGGNQQNYQDDNGSQYGGSNYSSHSGGGSQRGGDDRSRHKGSNQNNLSHQMGNMN
MGGNRQGMYSQNPNSRFPTNTENNLAGSAQNPRAPIPRQQLAHATQTFAERPKEFVFDKKAVGKKTQLWTNHALVHLPEQ
TIKLYAYNIEITQNRKVVNKREQAGPIFWEIMRANRTHYPGYKYFVFNDVNMMWSYDQLQRAEGDIILKKDKGGNPTTYL
NYKYTGRLDFGKTIREDQDRQLLSTLVDSIATARVRTDCRKFIVFKRCVFALNKDEDNTLTHELTNKFYHGIDSRLGISV
AIRLNLRAGITACYDIQHSIFTRSGYPLIRLLCEIINLDVITNDEFENEWDEKLQNAQMTESNRKEMDSILKKLRLRYTL
ETGIKVSDNGEENVPSVLMVNDRGKEFTYYKLTETSARDTIFTDQNGVDRTVAQYFLDAHHIRLRYPNLPCLQKKSSKKN
PAFIAFPMEFVSYSVNPARFEGHMSEKLKMSFIQQTSYSMEQRRAILNKIIGQEDIADCPPVVDNTDKYMEKYNLSIEKE
MLSVNATVLPVPTLVYGEGSKFTDTKHHGVWDALINDPPRKVLENSIFIRNPDPNAPKKKKRLLGSILNIISPHNNYNTL
DWDKIYYHRLMGALELAGQPVAWEGQGMAAIQGLTHFHQRQSPEALIPDFFLELKRTANHEDNNKYKESEDEELIPFVLV
VFQVRCSALKLNDGLNNDYNFMKYWGDNAAGIYTQGILTTSLSSINENPIYCKLTHLMVEKILGKIGTTHRKIERGGDHR
SWTKLTDPENPCLVLGVDVSHPSTQDRLTNDIRRLSIATVVGNIDIDCCEFRASSRIQDMGNETIVSFQFEVNKRINEFI
ANNNGKRPAHIIMYRDGIAESDFKRTLYEERINIENACLEIDPAYQPTITYIVATKRHHTRFFLKNPEEGHEFQNYNILP
GTLVEDTVTTNEYYDFFLTTQVGQTGLARPTHYYVLHDTWKPMVSFWPTITHAFTYMFCRSTSTVSLPAPLLYAHLAAKR
AKECLDGAWITAPLQNMSFDLKQYKDVCMLTSAINVNHGLDGMTFV

>Ctr-SAGO-1
MEELAKQLNNVKFEKNIVCGSNPLAAKIVEKNKSRGTPVSISTNLRRIDIEKNQPIYKYAVQVLFFYNKGDKEESVEISK
SVSKGPEHERDKAKCSKVYKKSVSQSPELQKGGPFFYDRQSCLYSFSRLKNDEIMCSLKGAEISSRPSFIRAEFKLSKVK
ESFQSTSNDISKTVNIRPAQADKTILEALNLIISGKPLEDPNVLTIGNCVHYLYDDSKIQMDRVNGSGGKSSAVGTSKSI
RTLEGKEKTPTLYMTTELKTTLFHPDNATLFQVLSDYSGFSPQMKCNNPWALSMKDFFLGVSCYVNYGKFKDLGDERVMI
KIKGFGLSAREQTFECDKKETTVFKYFKEKYNMSLQYPDLFTVLARGRDGKNQNIPVECLVLCNSQPVRTEQMSKDEQAK
LIRRNTDKVVTEVGLGANVQGLIRVSQPETVTGYVLPKPTIKFGGEKKVNWNDPNKRGPATDFNITRFSKPAKLLSWEIV
FDQNVPLDSAIGPLINTMKEMGMEVRDPQKTFIVGGKLRPIFENAVKNKRQMLLFITKSSNNYHQEMKCLEQEFDLITQD
IRFETALRLPQQQNTRKNIINKINIKLGGLNYEIDNEDLKNRLVIGFETSQKGGLGDAPIAIGFSANMSAHHMQFTGGYI
FVKRTSDVYGSIVQTVVKICMDEVIKNRGKPSSILVYFSGVTEGQYGLINEVYVQQIKEACLSIHANCKPHIHVIAASKI
HNTRLYKEDQRGFSNLEPGTVVDETIVNPLLNEWYTTSAVARQGTNKAVKYTLIFTTQPVPISVWERLTNDLAYDHQIVY
HPVSLPAPLYIAGSYSTRGAQVLAQRRAIYNAEGEFDFEATNEKFGVLNKKLFSTRFNA

>Ctr-RDE-1
MTNIEVRSESPVHCLKWLPRPTEKCDGNFYVKKVLLLVNWFKFTKNVYDRDYFEYDVKMEKIKTFKDRNGILQRKRTEIP
IPERSTLFWNHLRHEAKKNPFDIEEYVFDEKDTVYSVNRHEKELISSMVDPVKGNIKYIMRLTYRNTFRLNFSREDPTKD
EEANRSYKFLKVVMTQKVRYSPDVTEKVQMEFAKNFVYDRNTILCVPESFHDPNRFENSLQIATRIEAWFGIYIGVKELS
NGDPVLNFAINEKLFMNAPKMSLLDYLLLVIDPGTSNDDVRNEKKRELKEGNLTITVYSRKRIDQYLGNIKLKCSEVWDD
SVKRMAERHLTYIELSDKNSHEMMVESRVGRGKDVKTFHVPLFKIYEKNNKHIEFPMLPLVNVKSGSKIYCVPIEHLELH
EKPQRYKGWIDFAMQEKFIQACTRKPHNYKTKTIEMLQNLDFTAEELNFVQKFSLSADLKMITCTGKVLKEPKLVNRNNK
QVEMTPIIRGFKEKTLNVVPEKELCCALFVLRDDYDQYPCLDENEAYSFYDALISGCEARGIKIGTHENPRARSILYNAS
ENKYGFYKDVTLKNGARCFEAAANNAKNMFDDLPDKENKILIFIVVGDQKLDAYGFIKHYCDVTLGVASQFIRVVTVKKA
LRELQQEKTSRKIFFNLALKVNAKLGGVNQELNYSVNAETSPEEKEQRKNMPLTMYVGIDVTHPTAGSGIDYSIASIVAS
INPGGTLYRNQIIVQQEWNPGEIPTGKEKTDILEKKFVDLLKEFTKNNNNRFPDHIVIYRDGVSDSEMLRVTCDELRTLQ
SEVHRYLEDRGGTEKEPQYTFIIIQKRHKTRLFRKIADKRPENKEEAKQWEEDMKESDQSGILNPSSGTTVDKGIVSKYK
FDFFLASHHGDLGTTRPGHYTVMYDSMNMTKDQIYKMTYELSFLSARCRKPVSLPVPVYYAHLSCEKAKELYKCFKQNTL
GEYGSRPSREEIEKHLQTNMRYPGMSFA

>Ctr-VSRA-2
MPSKANKKKAAAERSAALKTEEATVQANAPSTPVSAALPVPTKAETPVTSAPIQTKPAETPKASAPIPVEISEYGQVALL
KRLSSTTKPERVTTNSFQMKIDDKKVYRYLVTITKKFTTMAGVKKTVELAGAEGSDRHRQTIFFNLVKDFLASHPRAPKH
WAYDGAATLYSVDKFSELNFHRESEGEWRDKNAFFSRGKEGSLTVSISLNNKEPVLSMKDILQEDLSAQTSCPTRQMLQI
ALIQEARKRGMAVVDNGKQIFGQGTPAPNERWVIEKNGVGAGVKIVEGEKNAGAAHVVLDFKRTRFFATVPLHDVRLDFA
DRAKASQYIKGLKANTVYSTQLVRMDGFSQLNLRDIKVPERGDLLTSAATLAGRREDQYSLNRPVVESTSYNKKQSKMIT
YSFPMENLVLAPNQKLGPKHGNPPECVAVHKRYGETLTVGAATGLLADNDTLKAFGVSIQRTPLSIDAVNVPIPDIQYQG
MMVKPNSGKWSIGRAKLIVPKNIQQILVLYNSSALSKHYDISKVIGRLVDKLHSMGALLGMRITRITPEDLYTTYRKSSV
CEAIDKKMDSLSKVKDVMVIYADESTQPTHGILKLKERQSGVVTQRLCLDKSLLLKQSRDRQADIGASTITNILFKLNVK
AGGWNHRVTHTQNDIKKFWGQTSKTLFISYDVCHSSGAKSYCKGETSEEPSTVGFAYNGTLDPEVIIGDFHYQLPRNEEV
DEAILRQRAGMMLNHYVDSRKTFPENIVILRDGVSEGQHKMVVDKEFTAIKQGIQTALKNIYNNKPFSMPKFALSIVTKR
HAHRLFKNDTKIENVKSMIAIDSGIVKKSANELIFVSHCPLKGTAQPILVNTLINESVFSNNNQLVNVLAALSCAHQKST
TIVSLPETVYAADEYAKRGADIFETYKMWHKGTLPTIEDNDGVVQYNWSLITRDLCYHQSAFKNRRIV

>Ctr-ALG-4
MSRRNATSFVDNTLTSSGSGSMSPPITSRAASGQASPLSSNGSLSPPNIVDDQGSVSYNSDSPRDLSPLLLSELACLNMR
EVVARPGLGTIGRQIPVKSNFFAVDLKNPKMVVIQYHVEVHHPGCRKLDKDEMRIIFWKAVSDHPNIFHNKYALAYDGAH
QLYTVARLEFPDEQGSVRLDCEATLPKDNRDRTRCAISIQNVGPVLLEMQRTRTNNLDERVLTPIQILDIICRQSLTCPL
LKNSANFYTWKSSCYRIPTAAGQALDLEGGKEMWTGFFSSAHIASNYRPLLNIDVAHTAFYKTRITVLQFMCDVLNERTS
KPNRNNQRGPGGPGGPGTPGGYRGGRGGARGGGYGNFGNRGGPPTNGGRDDFGGSGLTFTMDTLSRDTQLSSFESRIFGD
SIRGMKIRATHRPNAIRVYKVNSLQLPADKLMFQGIDEEGRQVVCSVADYFSEKYGPLKYPKLPCLHVGPPTRNIFLPME
HCLIDSPQKYNKKMTEKQTSAIIKAAAVDATQREERIKQLAAQASFSTDPFLREFGVAVSSQMIETTARVIQPPPIMFGG
NNRSVNPVVFPKDGSWSMDHQTLYMPATCRSYSMIALVDPRDQTSLQTFCQSLTMKATAMGMNFPRWPDLVKYGRTKEDV
CTLFTEIADEYRVTSTVCDCIIVVLPSKNSDIYMTVKEQSDIVHGIMSQCVLMKNVSRPTPATCANIVLKLNMKMGGINS
RIVADQITNKYLVDQPTMVVGIDVTHPTQAEMRMNMPSVAAIVANVDLLPQSYGANVKVQKKCRESVVYLLDAIRERIIT
FYRHTKQKPARIIVYRDGVSEGQFSEVLREEIQSIRTACLAIAEDFRPPITYIVVQKRHHARIFCKFPNDMVGKAKNVPP
GTTVDTGIVSPEGFDFYLCSHYGVQGTSRPARYHVLLDECKFTADEIQNITYGMCHTYGRCTRSVSIPTPVYYADLVATR
ARCHVKRKLGLADNTDCDTNSLSSSLASLLNVRTGSGKGKKSHASSVDDESFSLPDASSDQILQDCVSVAGDFKSRMYFI

>Ctr-SAGO-3
MESLTASMAGATVVEKKKLGLKPLAAKLPKNPSNQVVDVDTNIRKLVITKNQPIFKYSIKIEYFYRRLDGSECSVEFSKS
TKNGTGHEEDKDRCQRVYLMAAKKFAAGDASQFFYDRQASLYTLKNLKIGQEGKAFEFKNDISKRANFLKATLTIKPVED
SFQATTNDITRCISSCPGLSDKTLLEAMNNIVSGPAINNPEVITIGSCVHYLFHPEQYGIQTHSYSAGERYSAVGATKAV
KVMEGTDKQNPSLFLVTEIKDTLFHPDDKNLLDLFKTFQGFRTDMKANSPGAQNLLKACEGLDVYMDYGAGAEAQERMVV
SIKGFGEPAAQCFFETPTGKTNVVKYFQQTYNLRIQHPNLFTIEAKGPKGGKMNLPVEFLVVCNSQKVTTSQMQKNEQKD
MIKLSAAKPHDRRARTDQVVRAIGLGGKKINEFVDVEQPIRIKGVQLPYPKISFAGNQAAVLPNPSSKIPTDFNSAGKFL
EAVALQSWELCYIQGQEVRGLKEALVQEMARCGMIVKQPDVTLIVNGDLRSVFQNAKDRKRQMLFFVIPERSELHQKIKA
LEQEFDVLTQEVKKETADKFFKQPQTRQNVVNKTNMKLGGLNYHITSPYFTPDLLIVGFETASRGGSGDGPVSVGFAANM
MKHWQKYCGAYIFVPRSKDVFGPILKDTMIKIFQTARGNGRLKPSEIIVYFNGVTEGQYSMINEEYLPLIKAAWEQNTNG
SQPPKITLIASSKTHNERLYKNERGQLSNLTAGTVVDHTIVNPVLNEFYLASCVARQGTTKTVKYTLISTDQEKQSMQRL
ETLTNDLCYDHQIIFHPVGLPVPLYIAGRNSQRGSVVLHESGARVKDGKIDYEKTNEELGYSKKALFGTRFNA

>Ctr-SAGO-4
MKLGLKPLAEKQKPAKEEGRLVNIETNIRKISVDPNQRLYQYSVSIYFVLNREDGSEMVLEMSKSTRRAEEHEQDKERCR
DVYEKAVEQFVELREGGPFFYDCQSTLWALSKIPSEELEFSVTEGVCTRRNFIRADFLLKAVESVEISTSDILRTASRCP
GLADRRLLDALAIIISEEPHSRQNVMTDGNAIHYMINDGVGIRSLQFPEGNLSSSVGAAKSLRLLDGSNDTPGLFMTTEM
KTSLFHPTDEPLISVLKTFQGFHLTLSKDTETARRITEAMKGMILYLNYGPFVGLGRDGVIVKLTGFGLPPHEQTFTVGG
ERVSLYDYFWRKYGIRIESDQLMTVETVNKSGRKDYFPPELLLVYRHQKVTGERMINNESRDLIKLAAAPPHVRRETTDR
VVEEVGLSSSRTECISISNPTTTTGRVLEPARKYFGNEENPRSFFDPMSLKNWVIVFYEGEAVRGIDRILVEEMRKNGMV
VAPPIIDCIPRGQLDPVFIQAKQAQIELIVFITKKFYDIHDWIKAKEQEYDVLTQEIHFETAQKVLRGKKDTLQGIVNKT
NMKLGGLNYFVQSKHLPVDCFVLGFSHSQKSYSDSNIATIGFSSNSMNHSHKFAGGYVFVKRSKDLFGGVISDVLRKTLK
AMKKNREKPSKVVIYFHGLADSQIALANEVYSQKCLDCFSSLKSTYTPELTIIASTKSHNERLYASENGRIQNLEPGTVV
DTGIVSPVFNEFYHAGATTWQGTTKTTKYTVVYSSEVVDLQKIESLTNDLCQDHQIVFTPTSLPTPIYVATESAERGNKI
LRIRNGPILKENGEIDIDGTNEQYGYKNKAIGETRFNA

>Ctr-CSR-1
MQSGNSNRGRDTRGNDRGNSGRGGRGSTRGKRGGGQHQELKVGENSEPGGGRQKDHSNAVRGGRGSYRGRGGGLDQQDQN
HGGHGARGSNRGGRGGYNQVPQQDQRQNNDQGERSSHRGGRGAPDHSPRRGQYQNSGGRGGRGGIAQAPRGRTNDFRGEH
GRSSRGRGNSSRGGSNIVDNHSRPVETNERHNGMLSSNKENMDNPSKMNSNGNPRLAINIFGLELSERKIFRHVVQMKLL
DNKTKKDHILTTMSAKGRGNRASKQKDNFLLLKMLLDKWAAKRGSNNTPVFAYDGAQSLFTLEGISERMVIKKDDALQIA
DISEFLKDSVKFLSGDLEISCEPDHDKPFFVQTEIREWSDPRFYAYLDIVTSQGAIRSGKYLSQSKGLYVNTSHMTELRA
KWAVAAQGIHKGCRIVGSTGPLPVLELDPQSTQYYAPIPLSQMIQLAFPRDFNANRGVNPNMRLQRAVKLLLKDLKCNPF
YEDLRDWATNTITVSDIDYNAHKDNDYKLKYPNLRFPYLPAAQCGTGPSRRLMPLEYLKVLPYQSIDRRVLEEFELTPRA
NAPNERWQILQKHYDEFGFNNEVMIEFGVLICNDPFQNISEIDGERVLAPKISYADPVHVDDEKRDWKAQDKKFAKAAVI
DHLVFVLVAGYTRNFKDDIRATEYVARAFMQRCQDKGMTIRNYELQTHEGERDSEAFLTSVFKKLVTHPKYRDSSFIPFV
LFVSDDVPNIHECLKFEERMSDIPTQHVLLKNIRKIRDNMERKSGGGRRAYDLTLDNIVMKANIKCGGLNYTADIPRDIA
CWNDTPTFVMGMDVAHPDRLATREGNPSTVGLSCNAAEHPYSFIGDFLYTDPRREAIQDEILRKFTDQNVRNFAEIRGFP
KKIIIFRDGVSFGEESEALREVEIIEQTIISAAMSMGLRDYAPKVLAIVVKKRHHTRFYAKGGHNGMTPSNPLPDTSVGG
DIAEYGKRQIFIQAFRPVQGTAKVPSFLILRDDEEVSDEHISKMVCAVCSLHQLVNSPTSIPTPVYVAHELAKRGTGLFK
AYRFKNGELRDDWDTLTNQLSYSTLDRLSKVRVV

>Ctr-ERGO-2.1
MSGGRGRGGRGGSRGGCHDLSREMGRLNIYSQGNGRNPRSELQVGDLAGSALQPRTPIPKKNLIHNTQTFAERPDSWVYD
KKAVGTKTTLLTNHALVHLPKETVQLYCYNIEIFQNRKLVNKREQASPLFHDFVRRNRRDFPNRLDYIFNDVNLLWTSSK
LPKAEGSVGDNRSYMVYKYTTRFDFGQNIREDQDKQLLSTLVDAIATARVRSGCSKFSVFKKCMFMVGDGTADDQIEAPL
SFQLGQGFDARCGISIGVRVNLRAGVTACYDISHTIFSRPGYPLIRLLCELINHEPISNQDFEEVWDERLRSAVMTERNR
NELDRILKKMKLRYSLETAIVIDRDGNVTNLAVDRRPKEFKYYQLTETTAQDTRFQDQEGRWWSVAQYFLEASNIRLRYP
NLPCFVKKPTRQRPQFIAFPMEFISYVADPKRFEGQMDEKIKMEMIRQTAYTAPQRFALLQHIIEQKPIADVPAAVDNND
SYMTRHGLSIEKEMLSVKATLLPPPTVVYGEGSKFEDVNRIGEWEAITHDPIRKVLEGSIYIRNKDGPQLKKRLLCSVIH
ISSPENGKNQIYYDHACYHRLMNAIEEAGQPVAWENYEERYAAIHGVLDYCQGVDNPYSIVEFFQKIKANLDNKYKQDED
EILIPLVLLVFQIKCSTLKGHRDQWDNDYNFIKYLGDSEVGIYTQGILAENLCAIKETPAICKLTRLLVEKILGKVGTTH
RKLEREGDHKSWTKITDPKSPTLILGVDVSHPSPRDRMENDIRKLSVATVVGNIDFDCSEYRASSRLQESGEENIVRFRE
EITVRLNAFIQNTGVRPKHIVVYRDGICEGDFQRILYEERINIEQCCVDMDPGYQPTITYIVVSKRHHTRFFLKDLSEGH
ASQGFNVLPGTLVEDTVTTKDYYDFFINTQVGQLGVAHPTHYYVLHDTWKPMVSFWPTVTHALTYMFCRATSTVSLPAPV
FYAHLAAKRAKECLDGAWTVARLRNMRLSVDNFHDINQLTQAININTQLDGMTFV

>Ctr-ALG-1
MAAEINPTDTTNETANVASNESMSGGPQYLQGVMNTALPPQTPSNGSSSSSFLGSGGPIASATSSQVVPTSGATQQPPIP
SAQAAASTALQNDLEEIFNSPPQQAQSFGDGSQRQASLAPGAPIGTSGAGIGEPSNTIGGQLPGLPGQLQGGGGQSGVQF
QCPRRPNHGMEGRAILLRANHFAVRIPGGTIQHYQVDVSPDKCPRRVNREIISCLISTFSKYFVNIRPVYDGKRNMYTRE
PLPIGRERMDFDVTLPGDSANERQFSVSLKWVGQVSLSTLEDAMEGRVRQVPFEAVQAMDVILRHLPSLKYTPVGRSFFS
PPVITGAAAIASAAASPQSTASVSSGSHSAGQYHAESKLGGGREVWFGFHQSVRPSQWKMMLNIDVSATAFYRSMPVIEF
IAEVLELPVQALTERRALSDAQRVKFTKEIRGLKIEITHCGQMRRKYRVCNVTRRPAQTQTFPLQLETGANIECTVGKYF
FDKYRIQLKYPHLPCLQVGQEQKHTYLPPEVCNIVPGQRCIKKLTDVQTSTMIKATARSAPEREREISNLVRKAEFSADP
FAHEFGITINPAMTEVKGRVLSAPKLLYGGRTRATALPNQGVWDMRGKQFHTGIDVRVWAIACFAQQQHVKENDLRMFTN
QLQRISNDAGMPIIGNPCFCKYAVGVEQVEPMFKYLKQNYSGIQLVVVVLPGKTPVYAEVKRVGDTVLGIATQCVQAKNA
IRTTPQTLSNLCLKMNVKLGGVNSILLPNVRPRIFNEPVIFFGCDITHPPAGDSRKPSIAAVVGSMDAHPSRYAATVRVQ
QHRQEIISDLTYMVRELLVQFYRNTRFKPARIVVYRDGVSEGQFFNVLQYELRAIREACMMLERGYQPGITFIAVQKRHH
TRLFAVDKKDQVGKAYNIPPGTTVDVGITHPTEFDFYLCSHAGIQGTSRPSHYHVLWDDNNLTADELQQLTYQMCHTYVR
CTRSVSIPAPAYYAHLVAFRARYHLVDREHDSGEGSQPSGTSEDTTLSHMARAVQVHPDANNVMYFA

>Ctr-NRDE-3
MDIFDGIMNAMDKTRPPGPSKEKETVKAKPSRAAPSQPPAPSHSGRNATPPVQPGRNQTPPTHPPRYHTPPVMGLPTHGA
PPPGFGPAPGYGPVPGFGPGPGFGSSVAYGSPRGAPQGYPIPPGYPAPSGYPVQPGYPSPHGYPSPHGYPSPHGYPVPHG
YPAPHGYPGYGPPPGILIPHAPGIPRPPPQSSRSPPPPPPPRDEKPSHPIPPPPPPLEDQVPPPPPPPPKSNHPMVTSIK
IPTRVPKRELVEQNGPYAKRRNGPPNETNKDSFVQLRNESVQLNYWEFDTTQMEESIAKIFFSASIRFKNGKTFELSDGI
VAVGGDVNRQVRRMTQIMLFKKFLTKSRVFNSTFKPEMAAYDTAETIYVPQSAMFEHISEHYMTFEEEDFTSDEWKVVAQ
ITRRPEPQFEVKVKYIGQVFTRGEKSLTAENRPELVRCIETITSQCTHSNEFLLYSTGTFPLNGKITEIDGTTEIRSGFN
KSTRLVEGPNASPRMLISVDVVRSAFNTSMPLVKFVANKLADIANPIKGNFGGGRGGGRGGFRGGFRGDRGHYGGSNRYQ
DDRRSSYGDRRDDRHRNDRHRDDRHREDRHREDRHRDETDDRSSEDRHRSKVDPHTNYDYEAIRRLENDFRDRRFSKSAM
EALSKALKGLECLPIHLKDAKANRSVLIDCVHNMTSETMTFTLDKEDGTTCDINIVDYFKDKYNYKIKFPYLPLIGSKRR
KHMDFFPMELLFVVPGQRIKASKMSNEIQAQMTGRNSALPSQHISESKQALEMCLKLGDGHLEAFKIKSSSQPIKAMAKI
LVPPAIRFANTTYGIDPVRGVRFQTDGTFVRPARIGQVTIICYDEGFSRDVDQFCKAFVSQSERQGIRFDVPRERWMVVN
TSPDDAFTTRNIMKKSIEKNSTIVIGIVLEKRPEIHDVLKYYEEKLGMQTLQITVDTARKFFGGGGRQTIDNVLRKVNPK
CGGTNFFVEIQSSIHNRTLCRDVEQLQRKLYHKTQFIGFELSHSGARTTFDRQNGTFDGEPTVVGVSYSLKYSTQLGGFS
YFQDSRVHKLTRLEEKFGICIDAYQKATKRTPETIVVFRVGSGEGDYQQVIAEVEEMRKSSEKSGKIPKFVVVLTQRSSH
IRIYKEHINGTKAVEQNVVSGTCIDTFPSTHGHDEFVLCCQSPLIGTVRPTRYTILYNDAGWTKNEVMNVTYQLAFGHQV
SYAPPAVPDVLFAASNLAKRGHANLKTHTKVGEVRGRIEKAKEEFDNLDGAANQLLVDEYISKLSDELNDMTIKGRNFWA

>Ctr-NRDE-4
MDERFQRDERRDSDRLSHHRPSFGHSSSGSSRPYRSAEEEYRLKREKLEADNSEAPQAKRRYEEASPPDSFSPIGTIPVQ
LNSWFVDISRMDECIKKILFKTTLIMGQKSFDLSDGLSAISGDSNRQVRRRAQCLLFKTWFTKYSHLFHGSLNPAICAYD
AADTIYVAASNVIDKIPEDNCTLTETDFTMDEWRVINKISRRRDSKFEIRFGSVGELYTRGPRAMSIENRSEFTRCLETI
SNQILHTDEFLLYNSGTYPVNDGILSEPDSVTMIKSGFSKVSKVVEDPDGNIQAILTVDTASSPFYKSSSVLKFVTAKLE
EERNGPPRGGGGRGGFGGRGRGGRGGFGGGDRRDSYGRDDRRDSRGDRHHGDRRDDRRDDRRDDRRGDRDRHRDDRHRRD
DRHRDDRHRDDSDDQRDQEIIPDYDDRAVRELEKKYHDTRDDTGKYIQKISAALKGLECQPTHLVSSTANRCIIIDSIHD
GTSVSVNFLRERRGEDPVNINIVQYYLDTYDYKIKYPNLPVVAAKQRGHMSYFPMELLQIVPGQRIKSSKMTAEIQSHMT
GRNSSLPQQHVEEANRILRDYLKVGSRNPFFEAFKMSMKSSSPIKMQAQILAPPDIQFDAGSPYVMNHQGVRFKESGRFV
KPAKIKSVMIINYDKGFKTEQEFCQGLYRACSEQGIRFEQSTHDWRIEHTNPDNFSETERLMNKAKEKKITIVIGIVYDK
KPIIHDYMKYFEEFMGQLTLQITTDTANKIAFGRGGRQTIDNVIRKLNPKCGGTNFYVEVPHKINGRMVCNDPGAMHKKL
FKNTQFIGFELSHTGARTKFDIQKCIFDGDPTVVGVAYSLKHCSQLGGFSYYQDTRLHKLTKLEEKFKKCLKRYEKEADT
LPETIVIYRIGSGEGDHPQILKEVEEMRTSAEFYQPGYVPNFLVILAQRRSHVRIFPEKICGGKPMDQNVPSGTCVDTFG
SAHMEFVLCCQTPLIGTVRPTRYTVLVNEPKWSKNEVMNVTYQLSFAHQVSYAPPAVPNVLYAAENLAKRGHNNYKIHER
LVNIRDSSYQIKEKYGDTVPPEKIEEALCEKFINKVCKEINGMTISGRNFWA

>Ctr-PRG-1.2
MASGSGRGRGRGSGSNNSNSKTQEYFGTIQPDLFVRQAGEPKVGSSGRAQKCYANFIPIEMEKVDYSIYQYHVEFDPTVD
SKHMRERMLLHPNVTDEIGRYHVFDGMILYLTTEWQQNQEIEVPHPKTEDLIKVIFKQTNRFLLDNAQTINIFNTIIRRC
FDEMQLTQLGRHYFNSKDARTVREYNMSILPGYETAIRMYEEKLMLCVENRFKMVRRDSMLSLFRNEMQACQNNRMRVLE
KMNEMYGGTTIITLYNNKLHRFTRLDWSITPMSEFQKDGENITLKRYFKDQYDKDITVDDQPIIISEGKPKQPGEPPQIN
YIVPELCYPTGLTDEMRKDFRMMREIATHTRMSPQQRLQETRKLLGQFHENDKVRSCLQYWGIRLDEDLARVNARVLKSE
PLLAEGTKKYEGRNAEWARGIKECGVYRGSNMTNWIVVGPSSGNSGMLAQKFIGEAMRLGNTLRVATGDPMCVPITGVTP
NDYLDGVKRAIQQVNGEKVHMLVVLLVDDNKTRYDSLKKFLCVECPIPNQCVNLRTLAGKASDGGENKNFGSIVLKIFLQ
MICKTGGALWKVNIPLTDTMIVGYDLYHDSTLRGKTVGACVSTTTGDYTQFYSQTRPHENPTQLGNNLTHFVRKALKKYY
DENNNNLPTRLILYRDGAGDGQIPYIKNTEVKLVRDACDMVTERAAKMSNKEHKPIKLAFIIVTKRVNMRILKQGANANT
AINPEPGTVVDTVVTRPERMDFYLVPQFVNQGTVTPVSYNIIYDDTQLGPDKHQQLAFKLCHLYYNWQGTVRVPAPCQYA
HKLAFLTAQSLHGDSDEQLRDKLFFL

>Ctr-PPW-2
MARKPLLEAMPGPAPPIAVPPGAFPPPPIPPGPAPALPITSEHKTANDACKLRLRSLGMSPGVKIYPPPLLPGQAGAPIQ
VQTNVFGIEVYKEHEIFQYTVHIKADISPTKEVIFTKKGREDFVVTDRHEKCTSIFYYLLTKHDDFFHTADNTFVYDGQS
VLFSTLDLFADLQEGARKSRYYTLTGSELNNEDLKSLPCIKFEVAATKNPRIKFSQEDIGRRSCDANIESVNRSYHQILE
LALNQNSVRDTSRVVVFEHGKMFFFKPLEEGYSKYDCVDVGDGKQMMPGIKKTVQFIEGPMGRGQNNPCVVIDAMKVAFH
KEQPLNEKLNEVCSRILGDKFNEYDRERCVGVIKGLDVYTTYLGRRRHLKIEGLHHEGAETSRFRVSDSDKPTVKEYFFN
KYGITLKHPKASLILCKERGQVNFFPMELLTVTPNQRVKISQQTSAQSQMTTKESAVLPDVRERLIMTGKAAAKIDNDNK
TLSGLGVRVMEEPLVVPGRQMPMIKIAVNQTDAVHPQREGKWRFNQYTRPATPPRIWAMYCVGTPGTRFSIELLQKFGEE
YAGMFWEKGVKMPPPVELGLQSTADIEPKLLAAAKSGCKFVFIITDDSITTLHQKYKFIENSTSMIIQDMKLSKAMSVLS
QGKKLTLENVINKSNVKLGGTNYVFQDTKNQMDGSLIVGVGLSSPPPGTRFVLDSMHVLNPTIIGFTHNGKSSQEWTGDF
VLSPAGQETLAPIEDIITECITGYQQWHDLQLPKRIIVYRSGASEGNHPNIMAYEVPLALSAIRNFKATIQLIYIMVSKD
HTYRFFKPNLLAGIPSAPSATKTASASGSRSSIGGPGNARACDLNIGPGVMVDTGVTNPACKQFFLNSHVTLQGTAKTPL
YSVLYDETGASMAKLEELTYSLCHLHQIVGLPTSLPTPLYVANEYAKRGRNLWNETCKKDPSIRQISDEGSQLRQLTDAI
NYKASGDLIDRRINA

>Ctr-ALG-5
MEDQWLLSAIYDDGLVEKIRERRTNSSRNTSINEYLNFSPSSRCFDEFLFPVIKEPKAQFQLAAKPRESHIGRLIPLLSN
QFQISCSGSLVYQYKIQITPFIPSKKLNRKIVSSLKEQIGELEEFNLVFDGIDTIYTTKPIDIRKMNNVVINVKGVVNTK
ESPNRFSISFRFVDSFLLDTKITDSQEPGKKLQMMHAIDTIFRQTSSGAFHVVLQSFFSITPHLQHGHGLGWGTVNLGLG
REVCYGFYQNVTETFNGLTMNLDVATTTFYRPIALVEFLAEILEVPLATVTDGRCLSDAQKKKFNREITGLKVETRHCGS
PRRFRVARCTWKPMDCMRITINDSQKQQVQISMIDYFKNRYNIDLKQRHLPCVEVGRSRECVLPLELCFLVSGQRCIKKL
NEQQIANLIRATSRNANERKTAVLNLHDRIKINDDPHAARFGLRVENELMQLHGRVLPAPRLLYCHPNSKRQNCVTTPTN
GTWDMRGKNFYLGVEIRKWAVACFAEPSTISSEQIHAFVLNLRKVAREIGMPFVDNFCFCRYTNSDQTTSVLDYLNAEYP
DLQLVLCIVPGKSTVYGDLKRRGDLLGVNTQCVRTHNVAKNSPHTLSNLCMKINSKLGGTNVVISAPPSSVARDPVLFVG
CHLSRNPTGIPDGASSSSHSDTSIACLVGSMDGYPTRFAPVFRVQPRQANTIVDLGDMIREAILNFWNSTGFKPHKIVIY
RAGIQDVEEILQVELRALREACSSIDPAYQPGFTFIGLDVTHHTRLFAADDADQIGASRNVPAGTLVETGVTVNNLFEFF
LVSHAGIQGTSRPTRYIVMWDDNQMPPEEIHEMTYQLCHTQSRCTRSVSIPSPVYYAKLVAQRAKILMADENFDIDRFRK
RALSDGMLFT

>Ctr-VSRA-1
MPSKNNKKKAAAERSAKLAAEAPVEAPTAEVAELAVEPKAPEPKPKEVAPYKPPAPLERLTVVNPLSIVTNSFLMAIKNI
KVFRHDVKIVKYEEGRNEFDLAASQGSCRQRQAIFYDLIAEILGTRFKWAYDGAATLYTIDEKFNNESVHMESKDLTKEA
KEAIFPKGSEGSLTISFSSNTETRVLETRDLLEENLSNQTSCPVRQMLQIVLSQQARIGKMLIVDGGNEIFVKGSAPRDK
KWAIEMDGVGAGIKIVGGVKNDGAAHLVLEFKKTQFFAPIPLKDLGLDFNNRFSTAKFLKGMKLNTTYSSQIVTVSGLSD
MPMSAITYPGGKVLTDGAKLAGLKESAFNQQWPAVQSKNYNKKLKKQITYSFPIENLKVAPNQKLAPKHGNPPKCAYPDK
RFVETQRVGESTGLLSANSIIQGFGVTVQTTPVTVQAVTVPIPVIQYKGVTVTPDITKQAKWNIPAVNFIEPAKIPKILI
LYGSSEFAGKVDALQGPLKKTASGLGVTIGIISSVDLEQDYPDLSVAEAIEKKMESLKALKEKPLVIHVDRNTQQTHALL
KLKERQCQVVTQQLDVDKALKQNVPGWSTLQNILLKLNVKSGGLNHKVVPDPMISRLWGDSSKTLIISYDVCHSSGAKSY
RKGETSEEPSTVGFAFNGTSRPQEIIGDFHYQLPRKEEVNQGLLKQRAGFIMTSYVASRKGIPENIVILRDGVSEGQHMM
VVSNEFPAIKEGIQEALEAINKVNKANHAIPKFALSIVTKRHAHRLYQKKDEVISNVPPMIAIDTEIVKKSGNELIFVSH
CPLNGTVQPIIVNTLINENVFKSNAELVRLLAALSCAHQKSTSIVSLPETIYAADEYAKRGSDVFETYKAWMSQRGIALP
MIEEESQYDWDKITQELCYHTSVFKKIRMA

>Ctr-PRG-1.1
MASSLGRGRGRGSGSGTGNSDGRSQSYFGTIQPDLFVRQAGEPKVGSSGRVQKCFANFIPIEMEKVDYSIHQFHVEFDPT
VDSKHMRERMLLHPNVIEEIGEYHVFDGMILYLTNEWQQNQEIEVPHPTTGNLIKVIFKQTNRFFLDNAQTINIFNTIVR
RCFDEMKLTQLGRHYFNKKDGRDVREYNMRILPGYETAIRMYENQLMLCIENRFKMVRKDSMFALLKNEMMSSQNNRMRV
QEKMNEMFGGSTVITMYNNKLHRFTRLDWTINPLSEFTKDGEMITLKKYFKDQYNKEILHDDQPIIISEGKPKQPGEPPQ
INYIVPELCYPTGLTDEMRKDFRMMKEIASHTRMSPQQRLTETRKLISEFQGNENVRNCLNYWGIRFSDDLAVVNARVLK
SEPLHAEGTKKYEGRNAEWARGVKESGVYRGSNMTNWIVVSPSTGNSGMLAQKFIAEARRLGNTLRVQTGDPMCVPLQGI
SPNDYLEGVKSAIAKVRNEQVHMLVVLLVDDNKTRYDSLKKYLCVECPIPNQCVNLRTLAGKASDGGENKNFGSIVLKIF
LQMICKTGGALWKVNIPLTDTMIVGYDLYHDSTLKGKTVGACVSTTSGDYTQFYSQTRPHENPTQLGNNLTHFVRKALKK
YYDENNNNLPTRLILYRDGAGDGQIPYIKNTEVKLVRDACDMVTERAAKLSNKEHKQIKFAFIIVTKRVNMRILKQGSNS
STAINPEPGTVVDTVVTRPERMDFYLVPQFVNQGTVTPVSYNIIYDDTELGPDKHQQLAFKLCHLYYNWQGTVRVPAPCQ
YAHKLAFLTAQSIHGDSDEQLRNKLFFL

>Ctr-SAGO-2
MENKMENLALNDKKNTGPKLGLIPLAAKKPRNTEKGTKVLVETNIRKLAISPNQPIFKYAVQVNFVYGKADGTQVVIEMS
KSLRKGTEHDNDKARCQKVYNEAIKRYDTLRTGGPYFYDRQASLYTLNKLKTENIAFDVVEGISKRPNFKKAQFVLKKVD
ESFQSTSNDINKTVNPCPANADKTLLEAMNIIVSGPAFENKNVITIGSCVHYLIDTTGVEVPLKEYQEGCLYSGVGASKA
VKTLEGTDKKSASLFMTTEMKSTLFHPDWIPLLDVLRSFRGFTTNLKSNIPAAQRIEKACVGLDVVLDYGPHKGLQEDGI
VMKIRKFSTSAVETTFELEDKTKTTVFNYFKKKYGITLKYPDLFTVETKGKLGKINIPVELLLLCPSQTVTNDQMINNEQ
ADMIKLSAAKPNIRKTTTDCIAKKVGLASNSIYGFIKVEEPVKVEGMVLNKPKIAFAGNKLAVLNDPRAKFPTDFNLAGK
YFIAKELSNWAIVFIQGEEIKGLADQLVNEMKNNGMKASNPVETFIVRGDLESVFKNGKAANRQLLFFVVKSRYNYHQQI
KALEQKYDLLTQEVRAETAEKVFRQPQTRLNIVNKTNMKLGGLNYAIGSESFNKPGRLIVGFETSQRSGGNPDYPISVGF
AANMLDHHQKFAGGYVYVKRDKDIFGSIVKDTLITILKTTYKYRGKPTDILLYFNGVSEGQFALLNEAFSKHVKDACMFL
DQNYRPNFTIIASSKTHNERLYKSDKGFIVNLEPGTIIDHTIVSPVYNEWYHASAAARQGTTKTTKFTLIFTTQAAEPMW
NLQQLTNDLCYDHQIVFHPVGLPVPLFIAGRYSQRGAMVLAANQGPVYTNDVMDLEETNKLLGYGSKKLFETRFNA

>Ctr-WAGO-1
MPPVPPVPPMPPMPPVTVPPMTPMAPVPADQQQLHQKTGNDACIKRLQQLNVEPGPRMYLKPNEPGKLGKPVQIQTNIFG
IEVVKDTIVYRYMVHAKADVSPTKEAIFTKKGKEDFVVLDRHEKCCNIFFHAVEKYSEFFKVKDGNSVIYDGQSTLYTTI
DLFSDVLPTEKKSKVFEICGTDIDNIDLKSLPCISLEIYAPRRNSLEVSAKTLGQRTADHNIEANNREYTQFLELALNQN
CVRETSRFGCFEHGKVYFLKATEEGFDPRDCVDVGDGKSLLPGLKKTIQYIEGPFGRGQNNPSVVIDGMKAAFHKEQNVV
EKISSIIGKDCQNGISDYDREKAAQVIRGLDCFSTYTNRVRHLRVEGLHHDCAANSKFELPDGKSCTVQQYFQDKYKIQL
KFPKVNLLICKERGNKNYFPPELMTITRNQRVTIPQQTSQQSQKTTKECAVLPDVRQRLIITGKNAVNLNNENELLQSLG
IKVYPEPLMIKARELDGKELVYDKKTVSESGKWRAPPGSFNKPASFPELWAIYAVGTNNSRFSASDLNSFSSMFMEVMHK
KGLSVRGPSEASLLHADQVMTKLQKVADSKCKYVFIITDDNITHLHQKYKALEQRTMMIIQDMKMSKALSVCRDGKRLTL
ENVLNKTNVKLGGLNYTVNDSKKSMTDDQLVIGVGVSAPPPGTKYILEGKGHLNPQIVGFASNAIATHEFVGDFVLAPVG
QDTMTSIEDVLKTSIELFEKNRKTLPKRIIIYRSGASDGSHPSILAYEIPLARAVIHSYNADIKMIYIVVTKEHSYRFFR
DELRGSKATDMNIPSGIVLDNACTHPACKQFFLNSHTTLQGTAKTPLYTVLADDCNAPMDRLQELTFTLCHHHQIVALST
SIPTPLYVANEYAKRGRALWAERTEGAPIEAEGSETNRLKELTKELAYQQTDLNCKRINA

>Ctr-WAGO-13
MKGIIVEPTHLSAEYNRNVVIAGFSVLNAKEQKFIKKEGTEIKRITVVEYFKSKHNITIKFPDLPLIVTGKGKKKAYFPM
ELLKIVPGQRIKVKKESAVQKSLMAEKNVVRPRKYMKIITSKIWEAYCRNNPYLKSFGIKVNEKPIELEAKVIYPAELVY
DRKVFCQRDDETFQFLPPNKTKFFKPAEVQKIVVINFDDAIENPDDLKTFCEKLYTKCSDNGMTMTTQPDTWTRLSLPSE
EIGKLELRMRDFKRQNVTIIIGVTKEKKPTVHDVLKYFEATLGIPTLHIHIDTAKHFINERGDAKTVKNVIRKLNPKCGG
INFIVQLMEFYKANRIVPCSVKPVSEILFEETQFIAFEMIHEFAGPLFNQSQRTFDEEPTFVGCSYSLELATDLGGFNYL
QEFNQYKLKNVKSKLEECLNHYKTAVGRFPKTVVVFRTGSEDELDRVREEIVHMKKALEDKNIKLIVLIVEKTSHFRIFP
TGAQQNVISGTVIDDTITTPDFYQFYLVSQTVDKGTARPIKYTVAVNDPGWKFNVLYNLTYFLACGHQVSYQSPAVPNVV
YAAENLAKRCRNNFLTHRKLGKLETTITRVLANYDDLNERKHDKELDGHMEMSTPVGSEGPSTSTSEGSSTSTSAGSSTS
TSPASSSSGPPTGDAFREAEKCRVRTNLLKVDISKMPERVVRLSMETALCCGEDRIKLSDQHTSITGGLNTLNRRLTLFL
IYRNVLAKRSNIFGSDIHKYAYDCAKTIYAIEEAYKGDDEIKLTLEEKDFSAGEWKLICRTVRKPETRFEVVISANGFVY
SQGPDPQELITLIEAVSSEDITMDLQYSYHMFSFGPWYGPEADKTAEIVEKLYKEIRLMENGKVAMLFDFKYIPFYDEMS
VLKFLTLKYAKFKRISGIAPIQIPSNQGQWDLEQRGQRAQRGEQNPRNRSRSRSPLTPEEPAQEVTDYNLTEAIDVEAVF
KDKRYPEFVKDIEESMKGIIAKPTHLTGEYNHNIVITGFSKENAIGQKVFMKKSTNEQEEVTVFEYFEIKHNHTIIFPYL
PLIVTGSGERKAYFPMELLQIVPAQRIKAQKQSAALKSLITEKNTVLPREYMAEIKMLWEERWKNNRFLAAFGIKVGKKV
VSPYAKMLHPPAVLFKNDMVCSMKEGTVQFAPLEGTHFWKPATVGTVAVINFDGAIEDETKLQTFCEKLYNMCLDNGMTM
TTQPDTWTRLSLPSEEIGKLELRMRDFKRQNVTIIIGVTKEKKPTVHDVLKYFEATLGIPTLHIHIDTANHFINMIKGDE
TIQNVIRKLNPKCGGINFLVEPPESVNGQPVCSNIADVKQRLFSETQFIGFEMIHGSAETLFNRNQKTLVEEPTFVGCSY
SLELATDLGGFNYLQEFNEYKLKNVEDKIEECLNHYNTAVGRFPKTVVVFRTGAGEDDFKRVQEEVVDMKKVLEDKNIKL
VVLLVQKTSRIRIFPAGITGAQQNVKSGTVIDDTITSPGRNEFIMVSQTAKKGTARPIRYTEVVNEPEWTKDELYHLTYF
LAFGHQVSYQPPAVPNVVYAAENLVKRGRNNFLVHCELGNLETTITDVLEEHEDPNDREFDEHILDEINYTLNGHALVRR
NFWA

>Ctr-WAGO-15
MSSFRIPKKPRTDAAGSSSGGPSSSSQPPRISLPSGGPSTGKTFKEAGSTKVQTNMFRVDISRMPTRMVRLCMDTHLCGG
KVKIKLSDGLTSVSGGLNTTDKRLALRVIFRKIVSRHPEIFGTDLLKYTFDCATTIYAVDGAFKGGNDKLEETLRKEDFK
DEEWSQISRIIRRQATYFKVAISANGYVYTRGSDFEAVKNRQELTRLIEIASSEVLNTPDFLQYGSQTFPLKTRVTNKPD
ETSEIRMGFDKGVRLLDKGDIAMSIDNKQSPFYSATSVLKFVTSKYGEHVGIPGAAPQRRGDQRGREDPRGQRNRSRSRS
PRREHQETPLDYDIGQVNHFMNAFTSKRDPAVFRAIEEAIKGIFAEPIHLPKTHNHNIVITGFAKTNAKNTYFKLNEGQE
NEATINVEDYFFQHRERRLQFPLLPLIETGKGNRKTYFPMELLKIVPGQRIKAQKMSTVVQSSMTGNKATLPREHIQRIR
EVMTNSLRLDNNPHLKSFGISVNREPIKLDAQMIHPAEVLFKNRAVCPMKSGSVQFAPLKNTQFWKPATIGMVAVVDFDN
MQLDLTSFCKNLHEKCSINGLKMTTQPTSWRHFNLHSNDTENLKSEMSNLKKQNVTIIIGITKEKKPAVHDVLKYFEATL
GLQTLQIHVNTATHFIKNTGTQTVENVIRKLNPKCGGVNFVVEPPQSVNRQPVCSNITDVKRRLFGKTQFIGFEMTHGSA
RTLFDRSQGTFDGEPTIVGCAYSLELATDLGGFNYLQEMNEYKLKNLEEKFGKCLKHYETAVGHLPETVVVFRTGAGEGD
FKRVQDEIADMKKALEGRKIQLVVLVVQKTSHTRIFPENIEGTSALSQNVKSGTVVDGGITSFGRQEFILVSQTGQIGTV
KPIKYTVAANDPGWTKNELCHLTYFLAFGHQVSYQPPAVPHILYAAENLAKRGRNNFLTHKKLDKVGESIKKVLREHGDL
NDGDHQKELESFMVDDISKVMNAMALERKNFWA

>Ctr-WAGO-14
MSTRAVNTRRSTRLSARLSTSNSTFQEAGTCKVQTNLFKVDISEMPARVVRLSMETTMCCGEERIKLSDLHTSGGLSATN
RRIALRKIFRKVFYKNRDIFEGDMFMYSYDYATTIYAIEAAYKGGDTDVEVTLREGDFYDEEEWAQVSRIIGKPATQFNV
VISRDGFVYSQGPEFESIQNRQKLIRLIEVLSSEIQKSPFSIQCSIRTYAIETEDTNEADDTAQILMELYKEIRWLDNKE
LAMLMDYKYSLFYKEISVLKLLTSKHEEVTRIFAPNQIPRDQGQRDLEQREQRAHREAEDPRDPQERRQSRSRSPHSPAP
EQRAQEDPDYNVTKVIEAQAAFHNKRYPEVFKNIELSMKGITAKPTHLPEEYNHTVVITGFSDLNARQQKVFMNEDTNEQ
EEVTVFQYFERKHRHTIQFPLLPLIVTGSGERKAYFPMELLQIAPGQRIKAQNQSTALKSLMAEKNTILPRKYRKLIKNI
WYSGWRLHNHLFLNYFGIKVLRKTVDSCAKMIHPPAVLFKDDKVCPMKIGTVQFAPLKGTQFRKPAKVKEIAVLNFDHAI
EDPATLGTFCRNLHKMCINNGMNIPQQPHAWLQLFHESVREKLEETMSGLQRQNVSIIIGITKEETSITIPILLKYFEAI
LGIPTLQIHIGTANSFINMIEGDETVQNVIRKMNSKCGEINFMVEPPESVNGQPVCSNIADVRQRLFPETQFIGFETIHG
SPWTLFNQNQRTVEGEPTTVGCAYSLELATDLGGFNYLQEMNEYKMKNVEGHIEECLNHYKTAVGRFPKTIVVFRTGTGE
DDFNRVQEEVVDMKKVLEDKDIKLIVLLAQKTSHIRIFHAEITGEDACQQNVKSGTVIEDTITSPGRNDFILVSQTANSG
TARPIRYTEVVNEPGWTKNELYHLTYFLAFGHQVSYQPPAVPNVVYAAENLAKRGRNNFVFHRKLGKLETTITDVLEEHE
DPNDRELDGHILNEINNILNENALKRRNFWA

>Ctr-ALG-2
MEMPPSVDSSDSVPPTTQSIFGGDELSLEPVKKGISFRAPRRPNHGVEGRAIVLRANHFAVRIPSGTIQHYKVDVQPDKC
PRRVNREIINCLTRTFSRFFSHSRPVYDGKSNMYTRERLPFTQDPMSFTVNLPGESATDRQFEITIKYGGDISLAGLEDA
MQGRIEEVPYDAVQAVDVILRHLPSMKYAPVGRSFFSPPHLSPSEGGHHPESKLGGGREIWFGFHQSVRPSQWKMMLNID
VSATAFYRSMPVIEFLAEVLELPVQALAERRVLSDSQRVKFTKEVRGLKIEITHCGQMRRKYRACNVTRRPAQTQTFPHQ
LENGSSYDCTVLKYFADKYQMVLKYPHLPCLQVGQEAKHTYLPLEVCEIVSGQRCFKKLTDNQTSTMIKATARTAPERER
DISNLVRKAEFTSDPFVHEFGIAINPQMTEVKGRVLSAPKLLYGGRTHSTALPNQGIWDLRGKQFHTGIEVHHWAIVCFA
DQQHVRENDLRNFTQQFQRISRDAGMPIMGNPVFCKYAIGVEQVEPILKYLKQNYREIQLVVCVLPGKTPVYAEIKRVGD
TVLGLATQCVQAKNVTRTTPQTISNICMKMNVKLGGVNCILVPNVRPKIFRDSVIFLGADITHPPAGDSRKPSVAAVVGS
MDAHPSKYSATVRVQPNRKEIILDLASMVQELLLQFYHNTGFKPARIVLYRDGVSEGQFFNVLQYELRAIREACMMIEKG
YEPGITFIAVQKRHHTRLFTVDKKDQVGRAFNIPPGTIVDVGITHPTEFDFYLCSHAGIQGTSRPSHYHVLWDDNDMTAD
ELQQMTYQMCHTYVRCTRSVSIPAPAYYAHLVAMRARFHLVDREHESGEGSQPSGTSEDSTFSTMARAVQVHPAANSVMY
FA

>Ctr-WAGO-4
MSPPPAPPVQVPPMAVPPEVPLELLANLAITSDHIAGNDDCVKRMRELNLPVGEKVYPGTKAPGQAGTEIDIQTNLFGIN
VVNHKEIYQYTVNIKADVSPTKEVVFTKKGNEDFIVTDRHKKCCAVFYYALHKYDNFFHGSTSTFVYDAQSMLFSTVNLF
PGHRDGGTKTQNFAIDGAEVDHDDLKGLSCIKLEIYPTKNPSLRFSQEDIGRRSSDARIDSINRGYHQIIELALNQSCLT
DSSRCVVFENGKLFFFKPLEEGFAKEDCTEVGDGKQMMPGIKKTVHFVEGPCGRGQNNPSVVIDGMKVAFHKEQLVIEKM
KETTNPNGVCNGINDIERVRCTAVIKGLDCYSNYTGRVVHLKIEGIYHEGARTTRFELQDGKAISVFDYFRDKYNVVLQY
PDANLIVCKVKGKENVYPMELLTITPNQRVKITQQTSAQSQKTTKESAVLPDVRQRLIMTGKAAAGIGTENEVLRGLGIQ
VMDEPLMVKGRQLPPVKLMGHQGSSILPRDGKWKIGRYTRPANAPNIWALYCVGTQNTRFNMQQLKAFGDEFVTMFKAKG
VDLPPPAETQLVSVTDIEQKLNDAAGSDCKFVFLITDDSITSIHQKYKLIEKSRNMVVQDMKLSKALSVVTQQKRLTLEN
VINKTNVKLGGTNFTYLDSKNYLADNLVIGIGVSNPPPGTKFFLENKGALNPTVIGFANNSRNAQEFAGDFALGSPGQDT
LAAVEDIVTELINEYKKSHNNRVPKRVMVYRSGASDGNHGSIMAYEIPLALSAVHSFSKDIQMVYIAVSKDHTFRFFRPN
LHALTSSAAPASDSRGGPRGPANTGPKPWDLNIGPGLMLDSCVTNPACKQFFLNSHITLQGSAKTPLYTVLYDDTNAPMH
ALEEVTFSMCHLHQIVGLPTSLPTPLYVANEYAKRGRNLWNETTQSNPERRQEGAEREQLQKLTSSINYKSANMSACRIT
A

>Ctr-DCR-1
MVVRVRPDLQCFNPRDYQVELLDKASKKNTIVQLGTGSGKTFIAVLLLKEYGVQMFAPLDQGGKRAFFIVEKVNLVEQQA
KHIEVHTSFKVGQVHGQTSNDLWKSPEHCAKFMQANHVVVITAQCLLDLINHAFIRIQDLCVMIFDECHHALGSKHPYRL
IMAKYKELKKANEPIPRVLGLTASLIKEKVAPDKLAEQLNKLESVLDSVIETASDLVSLSKYGAKPYEAIVLCKDFVTNR
LSLPNHDAIIGLLADTEAFVKTTTLFHPDLDLDPRKVIKNALKTTISAFHILGPWAGWKASQMWEKELTKLTKSQILPDK
AMIFLDLARTTMITIKRLLEKEMRQIRRLAELERYVPHRIMRLFQVFEMFSPEFQEKRLNAEVPERLSAIIFVEQRYIAY
SLHVMIRHIRQWEPKFKFLGSDYVVGASGQNLANSDNQGLHKRQTEVLRKFQRNDINVLVTTSVLEEGVDVKQCNVVIKF
DRPKDMRSYVQSKGRARRAGSRYVVLVDQTDVSACDSDLKDFQQIEKILLSRHRTVNNPTEDDSEINLDNVDELMAPLVV
EKTQATVKMSNAIALVNRYCAKLPSDIFTRLVPQSTIIPTEENGVTKYCAELLLPINSPIKRAILLKNPMPNKKTAQMAV
ALEACRQLYIAGELDDNLLPKGRESIAKLLEHIDEEPDEYTPGMSAKVGSSKRKQLYDKKIARALNESHVEADKECFIYA
LELERFREAEPVLNPKKRIFQDPNEYEYCFGFLSTKQIPKIPSFPLFLRQGNMKVRLTVAPEKTRVTQSELEEIQLFHDY
LFTQVLQMCKTGNLELDVTNNAPLNTLIVPLNKSKESGTYAINMKYVTEVVANMENMPRIPTDEVRRQYKFNPDDYKDAI
VMPWYRNMEQPAFYYVADVLTELRPSSKFPDSNFDTFNDYFIRKYNLEIYDQNQSLLDVDFTSNRLNLLLPRVQSQPRRV
RSMSNSSMSSTPATQSDSKESTTSNSHSQRQILIPELMDKHPISATLWNVIAALPSIFYRLNQLLLTDELRETILIKAFS
RESNDTKLKETLEWSPLTYTTNYEEKQSVIVKKIQQLRELNQKSLEAQQKEQNDEEIKLEEGGEEEEWKIGVWDPELAVQ
SGVEISRKTENIEGEDTETVGLSQGLHDGNISDEDDDLPFVMHDYTAHFTGNPNENGSHQSWGGAAEIVPSGWGDIENDG
SDLDTQPIPFQIIGGSGKFNVQALMEDVGRVFEPSMSGISQQGGAAVVPPQQPPNTSASVSNASVSTATSKSRPFTREEE
KLRKIQEELLAKARERLEELEISEEKDSPRRIEETVDLEEFRDDLLLDKEEEETVVRPKTMDEEIEELKRGALIKESKDN
DTFKTDALERVNCEVLAVAANDLPPRPFSFEKESQTMHGRLLKDHDQDIVSHVDSEVAYGVSPCLLLTALTTSNASDGMS
LERFETIGDSFLKFATTDYLYHTLQDQHEGKLSFARSKEVSNCNLYRLGRKLGIPQLIFANKFDAHDSWLPPCYVPTYNF
KAPNTEDAEEKDKEIERILNGQAIEEKPENKTGWDIGENTAKSTADGIETINFPKQNRLVNEDISPLPYNLLTQQNISDK
SIADAMEALIGVHLLTLGPNPTLKVMSWMGLKVIQKDAKTDVPPPLLRFIDTPTNPNASTNALNNLWQQFQFAQLEEKIG
YRFKDRAYLVQAFTHASYSNNRVTGCYQRLEFLGDAVLDYMITRFLFEDVHQYSPGVLTDLRSALVNNTIFASLAVKYEF
QKHFIAMCPGLHHMIEKFVKLCADRNFDTNFNAEMYMVTTEEEIDEGHEEDVEVPKALGDVFESVAGAIYLDSGRNLDTT
WQVLYHMMRGTIESCCANPPRSPIRELMELEASKARFSKMERILESGKVRVTVDVGNNMRFTGMGRNYRIAKATAAKRAL
RYLHMMEEQRRQTYSISSEF

**Supplementary Figures**

**
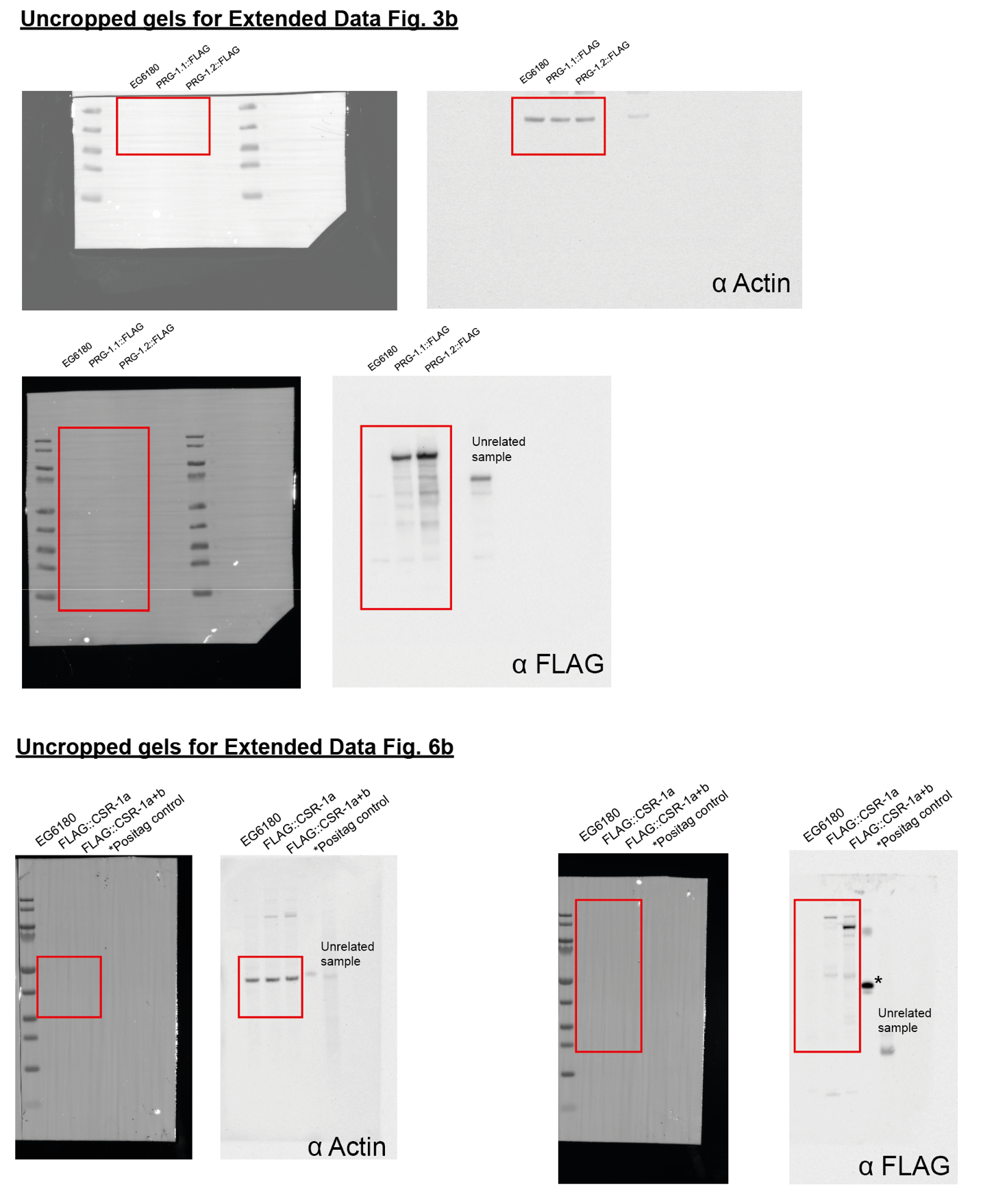
**

**SI Figure 1.** Uncropped gels for western blots shown in Extended Data Fig. 3b and Extended Data Fig. 6b.

**Supplementary Tables**

**Supplementary Table 1. Raw data for all crosses reported in this study**

|  | **Cross** | | | | | **Wild-type** | | | | | **Delayed** | | | | | **Other** | |
| --- | --- | --- | --- | --- | --- | --- | --- | --- | --- | --- | --- | --- | --- | --- | --- | --- | --- |
| **Figure #** | **Hermaphrodite** | **Male** | **Repeat** | **# F1s** | **# F2s** | **Total** | **NIC/NIC** | **EG/**  **NIC** | **EG/**  **EG** | **n.g.** | **Total** | **NIC/NIC** | **EG/**  **NIC** | **EG/**  **EG** | **n.g.** | **Sterile** | **EL** |
| 1b, 1c | QX2345 | EG6180 | 2 | 16 | 160 | **99** | 26 | 71 | 0 | 2 | **58** | 14 | 1 | 34 | 9 | 0 | 3 |
| 1b, 1c | EG6180 | QX2345 | 4 | 27 | 274 | **225** | 36 | 136 | 48 | 5 | **43** | 31 | 0 | 5 | 7 | 0 | 6 |
| 1c | QX2341 | EG6180 | 2 | 14 | 189 | **124** | 45 | 77 | 2 | 0 | **60** | 0 | 2 | 42 | 16 | 1 | 2 |
| 1c | EG6180 | QX2341 | 2 | 30 | 310 | **251** | 89 | 154 | 6 | 2 | **53** | 2 | 4 | 44 | 3 | 1 | 5 |
| 3b | QX2345* | EG6180 | 2 | 16 | 160 | **128** | 9 | 81 | 33 | 5 | **27** | 25 | 1 | 1 | 0 | 0 | 5 |
| 3b | QX2345** | EG6180 | 2 | 13 | 128 | **95** | 12 | 76 | 6 | 1 | **32** | 7 | 1 | 21 | 3 | 0 | 1 |
| 3d | INK266 | INK304 | 2 | 16 | 158 | **102** | 9 | 72 | 15 | 6 | **28** | 20 | 2 | 1 | 5 | 27 | 2 |
| 3d | INK264 | INK305 | 3 | 29 | 286 | **184** | 35 | 113 | 24 | 12 | **98** | 26 | 3 | 54 | 15 | 0 | 4 |
| 3f | INK559 | INK560 | 3 | 19 | 199 | **115** | 12 | 84 | 19 | 0 | **73** | 41 | 4 | 21 | 7 | 0 | 11 |
| 3f | INK555 | INK556 | 2 | 15 | 150 | **93** | 2 | 65 | 26 | 0 | **42** | 36 | 1 | 2 | 3 | 11 | 4 |
| 3f | INK640 | INK641 | 2 | 14 | 140 | **98** | 4 | 63 | 30 | 1 | **40** | 38 | 0 | 0 | 2 | 0 | 2 |
| 3f | INK822 | INK823 | 2 | 17 | 170 | **88** | 13 | 72 | 3 | 0 | **58** | 15 | 4 | 31 | 8 | 19 | 5 |
| 3g | INK769 | INK779 | 2 | 15 | 148 | **110** | 8 | 66 | 31 | 5 | **34** | 31 | 0 | 1 | 2 | 0 | 4 |
| 3g | INK772 | INK780 | 2 | 14 | 139 | **111** | 10 | 57 | 42 | 2 | **22** | 21 | 0 | 1 | 0 | 0 | 6 |
| 3g | INK757 | INK760 | 2 | 17 | 170 | **126** | 6 | 82 | 28 | 10 | **42** | 39 | 1 | 1 | 1 | 0 | 2 |
| 3g | INK763 | INK766 | 3 | 12 | 115 | **62** | 17 | 39 | 5 | 1 | **40** | 16 | 3 | 19 | 2 | 10 | 3 |
| Ext.Fig.6 | INK372** | INK351 | 2 | 15 | 237 | **147** | 42 | 99 | 1 | 5 | **73** | 18 | 3 | 31 | 21 | 7 | 10 |
| 4d | INK459 | EG6180 | 2 | 11 | 110 | **56** | 3 | 49 | 1 | 3 | **51** | 24 | 1 | 22 | 4 | 0 | 3 |
| 4d | EG6180 | INK459 | 2 | 11 | 110 | **83** | 5 | 48 | 30 | 0 | **22** | 16 | 2 | 1 | 3 | 0 | 5 |
| 4d | INK918 | INK459 | 2 | 14 | 140 | **88** | 7 | 69 | 7 | 5 | **50** | 18 | 0 | 31 | 1 | 0 | 2 |
| 4g | INK840 | INK112 | 2 | 15 | 190 | **148** | 60 | 69 | 6 | 13 | **32** | 0 | 2 | 26 | 4 | 0 | 10 |
| 4g | EG6180 | INK840 | 2 | 20 | 200 | **163** | 27 | 85 | 49 | 2 | **28** | 22 | 2 | 0 | 4 | 0 | 9 |
| 4g | INK918 | INK840 | 2 | 12 | 178 | **143** | 24 | 80 | 35 | 4 | **23** | 15 | 3 | 2 | 3 | 0 | 12 |
| Ext.Fig.3 | INK264 | QX2345 | 2 | 9 | 89 | **56** | 4 | 41 | 11 | 0 | **32** | 20 | 0 | 12 | 0 | 1 | 0 |
| Ext.Fig.3 | EG6180 | INK305 | 2 | 12 | 118 | **90** | 9 | 55 | 25 | 1 | **26** | 20 | 1 | 4 | 1 | 0 | 2 |
| Ext.Fig.3 | INK305* | INK264 | 2 | 28 | 279 | **213** | 11 | 132 | 59 | 11 | **58** | 45 | 2 | 3 | 8 | 0 | 8 |
| Ext.Fig.3 | INK305** | INK264 | 2 | 25 | 267 | **141** | 27 | 108 | 0 | 6 | **111** | 32 | 4 | 58 | 17 | 10 | 5 |
| **Figure #** | **Hermaphrodite** | **Male** | **Repeat** | **# F1s** | **# F2s** | **Total** | **KO/KO** | **KO/**  **EG** | **EG/**  **EG** | **n.g.** | **Total** | **KO/**  **KO** | **KO/**  **EG** | **EG/**  **EG** | **n.g.** | **Sterile** | **EL** |
| 5c | INK112 | EG6180 | 2 | 16 | 160 | **116** | 4 | 61 | 36 | 15 | **37** | 27 | 1 | 3 | 6 | 2 | 5 |
| **Figure #** | **Hermaphrodite** | **Male** | **Repeat** | **# F1s** | **# F2s** | **Total** | **NIC/NIC** | **NIC/KO** | **KO/KO** | **n.g.** | **Total** | **NIC/NIC** | **NIC/KO** | **KO/**  **KO** | **n.g.** | **Sterile** | **EL** |
| 4a | QX2345 | QX2361 | 3 | 19 | 190 | **134** | 43 | 57 | 0 | 34 | **55** | 1 | 3 | 31 | 20 | 0 | 1 |
| 4a | QX2361 | QX2345 | 2 | 14 | 140 | **101** | 34 | 59 | 1 | 7 | **31** | 1 | 1 | 23 | 6 | 0 | 8 |
| Ext.Fig.7 | QX2361* | INK459 | 2 | 20 | 200 | **192** | 52 | 72 | 34 | 34 | **6** | 0 | 1 | 1 | 4 | 0 | 2 |
| **Figure #** | **Hermaphrodite** | **Male** | **Repeat** | **# F1s** | **# F2s** | **Total** | **NIC/NIC** | **NIC/Δ** | **Δ/Δ** | **n.g.** | **Total** | **NIC/NIC** | **NIC/Δ** | **Δ/Δ** | **n.g.** | **Sterile** | **EL** |
| 4b | QX2345 | INK293 | 2 | 19 | 190 | **137** | 39 | 56 | 0 | 42 | **51** | 0 | 0 | 18 | 33 | 0 | 2 |
| 4b | INK293 | QX2345 | 2 | 13 | 129 | **119** | 27 | 59 | 24 | 9 | **5** | 1 | 3 | 1 | 0 | 1 | 4 |
| 4c | INK293 (inj. RNA) | INK460 | 2 | 65 | 650 | **535** | 112 | 252 | 67 | 104 | **90** | 4 | 7 | 61 | 18 | 1 | 24 |
| 4c | INK293 (control) | INK460 | 2 | 30 | 296 | **279** | 51 | 116 | 56 | 56 | **12** | 1 | 0 | 6 | 5 | 1 | 4 |
| Ext.Fig.7 | INK442 | QX2345 | 2 | 12 | 119 | **81** | 27 | 53 | 0 | 1 | **33** | 0 | 3 | 21 | 9 | 0 | 5 |
| **Figure #** | **Hermaphrodite** | **Male** | **Repeat** | **# F1s** | **# F2s** | **Total** | **AF/**  **AF** | **AF/**  **HK** | **HK/HK** | **n.g.** | **Total** | **AF/**  **AF** | **AF/**  **HK** | **HK/**  **HK** | **n.g.** | **Sterile** | **EL** |
| 1c | HK104 | AF16 | 2 | 14 | 139 | **110** | 7 | 70 | 32 | 1 | **29** | 28 | 0 | 0 | 1 | 0 | 0 |
| 1c | AF16 | HK104 | 2 | 6 | 64 | **51** | 1 | 37 | 12 | 1 | **13** | 12 | 0 | 1 | 0 | 0 | 0 |

* three generations following paternal inheritance

** nine generations following paternal inheritance

*** mothers are selfing individuals from the line INK372 that are csr-1(ko)

**Supplementary Table 2**. **List of strains used in the study.**

| **Strain** | **Short name** | **Species** | **Genotype** | **Description** | **Source** |
| --- | --- | --- | --- | --- | --- |
| AF16 | AF16 | *C. briggsae* | wild type | Wild isolate from Ahmedabad, Gujarat, India. Collected by A. Fodor | CGC |
| DL238 | DL238 | *C. elegans* | wild type | Wild isolate from Manuka National Reserve, Hawaii, USA | CGC |
| EG6180 | EG6180 | *C. tropicalis* | wild type | Wild isolate from El Yunque, Puerto Rico. Lat 18.3 Lon -65.8. Found in rotting fruit by M. Ailion and E. Jorgensen | Christian Braendle |
| HK104 | HK104 | *C. briggsae* | wild type | Wild isolate from Okayama, Japan. Collected by S. Baird | CGC |
| INK112 | *slow-2(-)/grow-2(-)* | *C. tropicalis* | *slow-2(abu70*[p.C71X]*)*; g*row-2(abu58*[p.7VX]*)* III; EG6180 | *slow-2 grow-2* double mutant in EG6180 background | This study |
| INK255 | Chr. III NIL *dpy* | *C. tropicalis* | *dpy(abu148)* X; *qqIR46* | dpy mutant in QX2345 NIL background | This study |
| INK264 | *prg-1.2(-)* | *C. tropicalis* | *prg-1.2(abu185*[p.Y183X]*)* I; EG6180 | *prg1.2* mutant in EG6180 background | This study |
| INK266 | *prg-1.1(-)* | *C. tropicalis* | *prg-1.1(abu183*[p.M189X]*)* I; EG6180 | *prg1.1* mutant in EG6180 background | This study |
| INK293 | slow-1(Δ)/grow-1(-) Chr. III NIL | *C. tropicalis* | *slow-1(abu122*[Δ*slow-1*]*)*; *grow-1.1(abu178*[p.T12SfsX2]*)*; *grow-1.2(abu132*[p.T12SfsX2]*)* III; qqIR46 | *slow-1* full coding deletion and grow-1.1 grow-1.2 mutant in QX2345 NIL background | This study |
| INK304 | *slow-1/grow-1; prg-1.1(-)* | *C. tropicalis* | *prg-1.1(abu173*[p.M189X]*)* I; *qqIR46* | *prg1.1* mutant in QX2345 NIL background | This study |
| INK305 | *slow-1/grow-1; prg-1.2*(-) | *C. tropicalis* | *prg-1.2(abu185*[p.Y183X]*)* I; *qqIR46* | *prg1.2* mutant in QX2345 NIL background | This study |
| INK348 | 3xFLAG::CSR-1a | *C. tropicalis* | *csr-1*(abu235[N1-3xFLAG::*csr-1*]) IV | FLAG::CSR-1a tagged strain | This study |
| INK351 | *myo-2p::mScarlet* EG6180 | *C. tropicalis* | *abuSi10[myo-2p::mScarlet::unc-54 3'UTR + HygR(+); abuSi9] IV* | EG6180 with red fluorescent pharynx. Single copy insert in Chr. IV abuSi9 landing site. Transgene used as a visual marker for crosses | This study |
| INK364 | *csr-1*::Neo/+ | *C. tropicalis* | *csr-1(abu239*[*csr-1*::*p.Leu337X::rps-20p::NeoR::rps-20* 3'UTR]) IV/+ | Balanced csr-1 null strain. Propagate on G418. Only hets and a few csr-1(-) survive | This study |
| INK367 | *csr-*1(-) | *C. tropicalis* | *csr-1(abu239*[*csr-1*::*p.Leu337X::rps-20p::NeoR::rps-20* 3'UTR]) IV/+; *qqIR46* | *csr-1* null strain. High embryonic and larval lethality but viable | This study |
| INK372 | slow-1/grow-1; csr-1::Neo/+ | *C. tropicalis* | *csr-1(abu239*[*csr-1*::*p.Leu337X::rps-20p::NeoR::rps-20* 3'UTR]) IV/+; *qqIR46* | Balanced csr-1 null strain in QX2345 NIL background. Propagate on G418. Only hets and a few csr-1(-) survive | This study |
| INK374 | 3xFLAG::PRG-1.1 | *C. tropicalis* | *prg-1.1(abu250*[3xFLAG::*prg1.1*]) I | PRG-1.1 FLAG tagged strain in EG6180 background | This study |
| INK377 | 3xFLAG::PRG-1.2 | *C. tropicalis* | *prg-1.2(abu253*[3xFLAG::*prg1.2*]) I | PRG-1.2 FLAG tagged strain in EG6180 background | This study |
| INK387 | 3xFLAG::CSR-1a+b | *C. tropicalis* | *csr-1(abu258*[N2-FLAG::*csr-1*]*)* IV | FLAG::CSR-1a+b tagged strain | This study |
| INK442 | *slow-1(Δpr)/grow-1(-)* Chr. III NIL | *C. tropicalis* | *slow-1(abu313*[Δprom::*slow-1*]*)*; *grow-1.1(abu310*[p.T12SfsX2]*)*; *grow-1.2(abu154*[p.T12SfsX2]*)* III; *qqIR46* | *slow-1* promoter deletion and grow-1.1 grow-1.2 mutant in QX2345 NIL background | This study |
| INK459 | mScarlet::SLOW-1 | *C. tropicalis* | *slow-1(abu287*[*mScarlet::slow-1*]*)* III; *qqIR46* | mScarlet N-terminal endogenously tagged *slow-1* in NIL background | This study |
| INK460 | *slow-1/grow-1; myo-2p::mScarlet* | *C. tropicalis* | *abuSi10[myo-2p::mScarlet::unc-54 3'UTR + HygR(+); abuSi9] IV; qqIR46* | Chr. III NIL with red fluorescent pharynx. Single copy insert in Chr. IV abuSi9 synthetic landing pad (SLP). Transgene used as a visual marker for crosses | This study |
| INK461 | mScarlet::SLOW-1 *dpy* | *C. tropicalis* | *slow-1(abu287*[*mScarlet::slow-1*]*)* III; *dpy(abu148)* X; *qqIR46* | mScarlet N-terminal endogenously tagged *slow-1* in NIL background and recessive dpy mutation in Chr. X | This study |
| INK531 | NIC203 *dpy* | *C. tropicalis* | *unc(abu308) X;* NIC203 | Uncoordinated mutant in NIC203 background obtained by EMS mutagenesis. Recessive allele. Backcrossed 4X into NIC203. Mutation maps to Chr. X | This study |
| INK555 | *21ur-06949(Δ)* | *C. tropicalis* | *21ur-06949*(abu384[Δ*21ur-06949*]) IV | deletion of piRNA 21ur-06949 | This study |
| INK556 | *slow-1/grow-1*; *21ur-06949(Δ)* | *C. tropicalis* | *21ur-06949*(*abu384*[Δ*21ur-06949*]) IV; *qqIR46* | deletion of piRNA 21ur-06949 in NIL background | This study |
| INK559 | *21ur-06949(Δ)*; *21ur-06917(Δ)* | *C. tropicalis* | *21ur-06949*(abu387[Δ*21ur-06949*]) IV; *21ur-06917*(abu387[Δ21ur-06917]) IV | double deletion of piRNAs 21ur-06949 and 21ur-06917 | This study |
| INK560 | *slow-1/grow-1*; *21ur-06949(Δ)*; *21ur-06917(Δ)* | *C. tropicalis* | *21ur-06949*(*abu387*[Δ*21ur-06949*]) IV; *21ur-06917*(*abu387*[Δ*21ur-06917*]) IV; *qqIR46* | double deletion of piRNAs 21ur-06949 and 21ur-06917 in NIL background | This study |
| INK640 | *21ur-06917(Δ)* | *C. tropicalis* | *21ur-06917*(abu470[Δ21ur-06917]) IV | deletion of piRNA 21ur-06917 | This study |
| INK641 | *slow-1/grow-1; 21ur-06917(Δ)* | *C. tropicalis* | *21ur-06917*(*abu470*[Δ*21ur-06917*]) IV; *qqIR46* | deletion of piRNA 21ur-06917 in NIL background | This study |
| INK735 | slow-1/grow-1; 3xFLAG::PRG-1.2 | *C. tropicalis* | *prg-1.2(abu253*[3xFLAG::*prg1.2*]) I; *qqIR46* | PRG-1.2 FLAG tagged strain in NIL background | This study |
| INK757 | *set-25(-)* | *C. tropicalis* | *set-25*(*abu471*[p.I543X]) III | set-25 mutant in EG6180 background | This study |
| INK760 | *slow-1/grow-1*; *set-25(-)* | *C. tropicalis* | *set-25*(*abu472*[p.I543X]) III ; *qqIR46* | set-25 mutant in NIL background | This study |
| INK763 | *set-32(-)* | *C. tropicalis* | *set-32*(abu475[p.F232L;N233T;Y234N;T235X]) IV | set-32 mutant in EG6180 background | This study |
| INK766 | *slow-1/grow-1*; *set-32(-)* | *C. tropicalis* | *set-32*(abu475[p.F232L;N233T;Y234N;T235X]) IV; *qqIR46* | set-32 mutant in NIL background | This study |
| INK769 | *wago-11(-)* | *C. tropicalis* | *wago-11*(abu478[p.H369T;L370D;P371X]) II | wago-11 mutant in EG6180 background | This study |
| INK772 | rrf-1(-) | *C. tropicalis* | *rrf-1*(*abu481*[p.I501T;Y502D;V503X]) I | rrf-1 mutant in EG6180 background | This study |
| INK775 | slow-1/grow-1; 3xFLAG::PRG-1.1 | *C. tropicalis* | *prg-1.1(abu250*[3xFLAG::*prg1.1*]) I*; qqIR46* | PRG-1.1 FLAG tagged strain in NIL background | This study |
| INK779 | *slow-1/grow-1*; *wago-11(-)* | *C. tropicalis* | *wago-11*(abu478[p.H369T;L370D;P371X]) II; *qqIR46* | wago-11 mutant in NIL background | This study |
| INK780 | *slow-1/grow-1*; *rrf-1(-)* | *C. tropicalis* | *rrf-1*(*abu481*[p.I501T;Y502D;V503X]) I; *qqIR46* | rrf-1 mutant in NIL background | This study |
| INK822 | *21ur-06949(Δ)*; *21ur-06917(Δ)*; *prg-1.1(-)* | *C. tropicalis* | *21ur-06949*(abu387[Δ*21ur-06949*]) IV; *21ur-06917*(abu387[Δ21ur-06917]) IV; prg-1.1(abu183[M189X]) I; EG6180 | double deletion of piRNAs 21ur-06949 and 21ur-06917 and prg1.2 mutant in EG6180 background | This study |
| INK823 | *slow-1/grow-1*; *21ur-06949(Δ);* *21ur-06917(Δ)*; *prg-1.1(-)* | *C. tropicalis* | *21ur-06949*(*abu387*[Δ*21ur-06949*]) IV; *21ur-06917*(*abu387*[Δ*21ur-06917*]) IV; *prg-1.1(abu173[M189X])* I*; qqIR46* | double deletion of piRNAs 21ur-06949 and 21ur-06917prg1.2 mutant in NIL background | This study |
| INK840 | *mScarlet::*SL2*::slow-1* operon | *C. tropicalis* | *slow-1*(*abu425*[*mScarlet:*:SL2::*slow-1*]) III; *qqIR46* | An intergenic SL2 trans-splicing sequence from C. tropicalis gpd-2::SL2::gdp-3 was introduced in between mScarlet and slow-1 (INK459). This transformed the endogenous slow-1 locus into an operon | This study |
| INK918 | *rps-20p::mScarlet* [germline (+)] | *C. tropicalis* | *abuSi44*[*rps-20*p::*mScarlet*::*rps-20* 3'UTR + *HygR*(+); *abuSi43*] V | A single copy mScarlet insertion into EG6180 Chr. V synthetic landing pad (SLP). mScarlet is expressed in soma and germline | This study |
| N2 | N2 | *C. elegans* | wild type | Reference strain | CGC |
| NIC203 | NIC203 | *C. tropicalis* | wild type | Wild isolate from Capesterre Belle-Eau, Guadeloupe. Lat 16.05 Lon -61.63. Found in rotting flowers by N. Poullet and C. Braendle | Christian Braendle |
| QX2341 | Chr II NIL | *C. tropicalis* | *qqIR45 (II:8.0-8.7 Mb; NIC203 > EG6180); EG6180 Mito* | NIL carrying NIC203 TA element Chr. II in a EG6180 background | REF 9 |
| QX2345 | Chr. III NIL | *C. tropicalis* | *qqIR46 (III:10.42-10.46 Mb; NIC203 > EG6180)* | NIL carrying NIC203 TA element Chr. III in a EG6180 background | REF 9 |
| QX2355 | EG6180 dpy | *C. tropicalis* | *dpy(qq104)* X*;* EG6180 | Spontaneous dumpy recessive mutation in EG6180 background. Mutation maps to Chr. X | REF 9 |
| QX2361 | *slow-1(fs)/grow-1(-) NIL* | *C. tropicalis* | *slow-1 (qq100*[p.H125RfsX15]*); grow-1.1(qq101*[p.T12SfsX2]*); grow-1.2(qq101*[p.T12SfsX2]*)* III*; qqIR46* | *slow-1 grow-1.1 grow1.2 mutant in QX2345 NIL background* | REF 9 |

**Supplementary Table 3. Primers used for genotyping crosses**

| **Marker or Gene** | **FW primer** | **RV primer** | **Sequencing primer or gel** | **Comments** |
| --- | --- | --- | --- | --- |
| NIC203 Chr. III TA | GCCTAGAAAAACAATTGATGGCC | CGAGTAATTTCACCGGGTTG | gel | Deletion in EG6180. 699 bp amplicon in NIC203 and 556 bp in EG6180 |
| *grow-1.1* frameshift | AAATAGGCGGGGCTTTCTTA | GGAGGCAGGAGAGTCCTTCT | ACCGGTAAATGGTCGAATTCAGC | 1125 bp |
| *grow-1.2* frameshift | TGCTGAGGAGTCTTCGATTCCC | ATCTCATTTTCCCGCCAAACGC | ACCGGTAAATGGTCGAATTCAGC | 1085 bp |
| *slow-2* frameshift | AAGCCAATATGGAGTTGACGCC | TAACGGAGGCATCTGTGGATCG | GCGTATCATGCGAACTCTCCAA | 458 bp |
| *grow-2* frameshift | TCTCGAGAATTTCTGCACTGTTCAA | TGTACTGCATCCTCCGACGTTT | TCTCGAGAATTTCTGCACTGTTCAA | 655 bp |

**Supplementary Table 4. gRNAs used in this study**

| **Gene** | **Modification** | **Sequence** |
| --- | --- | --- |
| *slow-1* | *slow-1* deletion | ATTTTCCTGGGAATCAACAC |
| *slow-1* | *slow-1* deletion | TCCGTGTCAGCACAATATAT |
| *slow-1* | *slow-1* promoter deletion | AAATCCTCATTTCCCGTCAA |
| *slow-1* | *slow-1* promoter deletion | CGTGAATGCAGGAAACTCGC |
| *slow-1* | *slow-1* promoter deletion | AAAACCATTGACGGGAAATG |
| *slow-1* | *slow-1* promoter deletion | AAGTGTAGAATTTGCAGAAA |
| *slow-1* | mScarlet::*slow-1* | AGAGATCACAGAGCGTTACAA |
| *slow-1* | mScarlet::SL2::slow-1 | ATGGACGAGCTCTACAAGGG |
| *slow-2* | *slow-2* frameshift&stop | TTGTCACAAGAAATTCGAGT |
| *grow-2* | *grow-2* 3xStop | TATTCATTGATTCTATGCAA |
| *prg-1.1* | *prg-1.1* frameshift&stop | TATAACATGAGAATTCTCCC |
| *prg-1.2* | *prg-1.2* frameshift*&stop* | CTTTTGAGTTGAAGTAGTGA |
| *prg-1.1* | FLAG::*prg-1.1* | GTAAATATGGCTTCCAGTTT |
| *prg-1.2* | FLAG::*prg-1.2* | TACCGGATGCCATTATTACC |
| *csr-1* | neoR insertion | AAGAACGGTTTATCATGATC |
| *csr-1* | neoR insertion | CATCTCAAGGAGCAATCAGA |
| *csr-1* | FLAG::*csr-1a+b* | AACCGTGGACGAGATACTAG |
| *csr-1* | FLAG::*csr-1a* | CCGTTCGAGTTCATTTTTGA |
| *21ur-06949* | *21ur-06949* deletion | AAGGAAACAGAAAAATACATT |
| *21ur-06949* | *21ur-06949* deletion | CCAGTTGGGGAACCATTCTTCG |
| *21ur-06917* | *21ur-06917* deletion | TTCATAGTAACGATTTCCGG |
| *21ur-06917* | *21ur-06917* deletion | TTTAATCTTACCAATAACAA |
| *rrf-1* | *rrf-1* frameshift&stop | CCCCACACGAGTAATCTACG |
| *set-25* | *set-25* frameshift&stop | CGTTCAATGAGCTGATCAAA |
| *set-32* | *set-32* frameshift&stop | ATTCACATCAGTGTAATTGA |
| *hrde-1* | *hrde-1* frameshift&stop | AGAAGTTGAGGAGGCCCAAA |
| *wago-11* | *wago-11* frameshift&stop | TGAGTCTTTGGCAAGTGAAT |
| *mut-16* | *mut-16* frameshift&stop | AGATATCAATAAGTGTCTGA |
| *simr-1* | *simr-1* frameshift&stop | AGGATTGAGTGTGAACGAAG |
| Chr. V SLP | *rps-20p::mScarlet::rps-20 3'UTR*  [germline (+)] insertion | AAAGTCCACAATCTCCACGT |
| Chr. IV SLP | *myo-2p::mScarlet::unc-54 3'UTR*  insertion | AAAGTCCACAATCTCCACGT |

**Supplementary Table 5. Homology-Directed Repair templates used in this study**

| **Gene** | **Modification** | **Sequence** |
| --- | --- | --- |
| *slow-1* | *slow-1* deletion | CTATCTTCAAGGGGTCGGGGCCTAGGAAAAGTGGGCGGAGTTTGAAATTTAAATAGGCGGGGCTTGTGGAGAAAGTGGGCGTGGCCAGTGTATTGGCGGTAATTCAAATTCCGTTTCTTTTGTTTCCCATTTTTCTTGATTTTTCTCCGACAAAAATTACTTTTTTGAGTCAGAAATGAT |
| *slow-1* | *slow-1* promoter deletion | GCGAATTTCTTGAGTTTTCAAGTGATTTAGAAACAGAAACACGTAGAAAAGTGTAGAATTTGCAGAAACCAGATGAAATGGAGCTTGTAACGCTCTGTGATCTCTCAAAGCTTGCAATTCTCCACGAAACACTGC |
| *grow-2* | *grow-2* 3xStop | CTCTCTCTCCCTCACTCATCTAGTCCCCTCCTTGCGCGTATTCATTGATTCTATGCAATGGGAAACAACGCCGAATGACTAATTGAAAGAAATTAACGAATCAATAAAACAGACAACGGTGATCTTTCAGAAATTCCTAATA |
| *prg-1.1* | FLAG::*prg-1.1* | GGACTTTATTTCTTTGTAATTATCAACTTAATTACATCAATAATTTTTTCAGGTAAATATGGATTACAAAGACCATGATGGTGACTATAAGGATCATGATATTGACTATAAAGATGACGATGACAAGGCTAGTTCCTTAGGTAGAGGCAGAGGACGCGGCTCTGGGTCAGGAACTGGAAACAGTGATGG |
| *prg-1.2* | FLAG::*prg-1.2* | GGAGTTGCTGTTGTTAGATCCAGATCCACGTCCTCTGCCTCTTCCACTACCGGATGCCTTGTCATCGTCATCTTTATAGTCAATATCATGATCCTTATAGTCACCATCATGGTCTTTGTAATCCATTATTACCTGGAAAAAATATGCGAAAAATCTAGGAGAAACCGAGGAGAATCGACGG |
| *csr-1* | FLAG::*csr-1a+b* | GCGGGTTTCGAGAAAAGTAACAATTTTCAGCACAAAATGCAGTCTGGGAATTCTAACCGTGGACGAGATTACAAAGACCATGATGGTGACTATAAGGATCATGATATTGACTATAAAGATGACGATGACAAGACTAGGGGGAATGATCGTGGAAATAGTGGAAGAGGTGGACGTGGATCGACTAGAGGTAAAAGAGG |
| *csr-1* | FLAG::*csr-1a* | GCATCTTCACACCTAGCTTAACTTCAGATAATCCGTCAAAAATGGATTACAAAGACCATGATGGAGACTATAAGGATCATGATATTGACTATAAAGATGACGATGACAAGAACAGTAATGGTAACCCCAGACTGGCAATTAACATTTTTGGACTTGAGCTTTCCGAACGCAAGATTTTCCG |
| *21ur-06949* | *21ur-06949* deletion | GGATGGAATGCGGTTTGAGAGACTATTGGAATCTTTAAATTTGAAGGAAACAGAAAAATGGTTCCCCAACTGGGAAATGCGTCATTATCCAAGCAGAGGAGTTCCCAACG |
| *21ur-06917* | *21ur-06917* deletion | CAGATTTTTTCAATGTTTCGCCATTGTCTCAAATTTTCAAGGAAACAACGGCAAAAAATATTCTAACTAAATGAAAATAAAAAAAATGTGTTTTTGAACATTTC |
| *rrf-1* | rrf-1::stop | GGCTATCAGATTGTTCGTAAAGTGATCTTCACCCCCACACGAGTAACTGACTAACGTGGCACCGGAGACTATAATGGGAAATCGTGTGCTTCGGAAGTTCG |
| *set-25* | *set-25*::stop | CGCATGTGCCGAAAAAGAATTGAAAACAAGACGTTCAATGAGCTCTAACTAATGGAGGAATATCGGATGCTATCGGAATATGAAGACTATCC |
| *set-32* | *set-32*::stop | GCTAGCTCTCTTTCTGTTACCTGATTGATTATATTCACATCAGTTAGTCAAGGTCAAAGTTTTCGGAATCGTGAGATTCTCTAGGGTCTCACCC |
| *hrde-1* | *hrde-1*::stop | GGTGGCGATTCCGCTCGACAGTTCGATCCGCGAGAAGTTGAGTAAGTAAGGCACTTCAAGACAACAGAAATCTTCTCAACTCAATCGAGTTCGG |
| *wago-11* | *wago-11*::stop | GGTCTTTGCGAATCCAGTAATAACAATATTATGATTGTGAGTCTTAGTCAGTTATCGGCTCCGCAAATATACCCTTGATAGCCTCCTCAATGGCTCTG |
| *mut-16* | *mut-16*::stop | CCTTACCGATGACAATTGTGGCATATCCTTCATAAACTCTTGGCTTCCCATGAGGATGATTGTAAGACTCTCAAGACAATATCCAATATCAGTCAGTCACTTAGATATCTTCCGGTTCGAATCCATTCTCAAGGAATTCTACTCCAGC |
| *simr-1* | *simr-1*::stop | CAATACAGTTCCAGGAGTGATTTCTGCTTCGGTAGGATTGAGTTAGTCAGTTAAGGGGCTGCACTCGCAAGCGCTTCATTCAATTTGTCATCAGG |
| slow-1 | *mScarlet::slow-1* | TAATACGACTCACTATAGGGCGAAACGGAATTTGAATTACCGCCAATATATTGTGCTGACACGGAGACGTCAAATTGCGTACTTGTGCATTAAACCTCACGCGCGCTCCGATTTTCTCCTATTTTTCTTGTTTTTCCTGCCGTTTTTTCGCTTTTTTTCGCGATTTTTCGCATCAAAAATAGGAAAACAGGTTTTAAATACTTTTTTAAACGTATAAATGACTAAAATTATCCGAAATTTCGATACTGTTTCTCAAAAATCCTCATTTCCCGTCAATGGTTTTTTTTACAGATAGACAGATGAAATGGTCTCCAAGGGAGAGGCCGTCATCAAGGAGTTCATGCGTTTCAAGGTCCACATGGAGGGATCCATGAACGGACACGAGTTCGAGATCGAGGGAGAGGGAGAGGGACGTCCATACGAGGGAACCCAAACCGCCAAGCTCAAGGTCACCAAGGTAAGTTTAAACATATATATACTAACTAACCCTGATTATTTAAATTTTCAGGGAGGACCACTCCCATTCTCCTGGGACATCCTCTCCCCACAATTCATGTACGGATCCCGTGCCTTCATCAAGCACCCAGCCGACATCCCAGACTACTACAAGCAATCCTTCCCAGAGGGATTCAAGTGGGAGCGTGTCATGAACTTCGAGGACGGAGGAGCCGTCACCGTCACCCAAGACACCTCCCTCGAGGACGGAACCCTCATCTACAAGGTAAGTTTAAACAGTTCGGTACTAACTAACCATACATATTTAAATTTTCAGGTCAAGCTCCGTGGAACCAACTTCCCACCAGACGGACCAGTCATGCAAAAGAAGACCATGGGATGGGAGGCCTCCACCGAGCGTCTCTACCCAGAGGACGGAGTCCTCAAGGGAGACATCAAGATGGCCCTCCGTCTCAAGGACGGAGGACGTTACCTCGCCGACTTCAAGGTAAGTTTAAACATGATTTTACTAACTAACTAATCTGATTTAAATTTTCAGACCACCTACAAGGCCAAGAAGCCAGTCCAAATGCCAGGAGCCTACAACGTCGACCGTAAGCTCGACATCACCTCCCACAACGAGGACTACACCGTCGTCGAGCAATACGAGCGTTCCGAGGGACGTCACTCCACCGGAGGAATGGACGAGCTCTACAAGGGAGGTGGATCAGAGCTTGTGACACTCTGTGATCTCTCAAAGCTTGCAATTCTCCACGAAACACTGCAAAAAGTGCTCGACGTGTCTTGCCAAACCGAAGAAGAGATAATCAAAAAAGAAGAAGGAGTTCTTCGAGAAATCACCGCCGCCGACTGCAAGCAGATGGAGTCCCTGGAACGAATCGCATTTAATTCGTTTGTAACGAGTTTCGACAGGTTCAACGAGCTACCGGAACTGGATAAAGAAACTCTTATCAAACAGTACAGGACGTCTATGATGGCGCTCAATCGCCTATTGGAGGGATCAAGCTTTTGTTCCCTTTAGTGAGGGTTAATT |
| slow-1 | *mScarlet::*SL2*::slow-1* | CCGAGGGACGTCACTCCACCGGAGGAATGGACGAGCTCTACAAGTAACGGTTTGTTTCTCAAACAATGCAATTAGTCAACTGCTGATATTTAACACCTTCTTCCTTTTGTATTAGCCTCTAATGTTCCAATAGAAGCTTACAAGTATTTGAATGGTCTCTTCACAATAAAGGTTGTATATTGCTGTCATTTACCGATTCATTGTCCATTCCACTCGTTCAATCGAGAATTATTATCTTATTGTTATGTTTCCTTCTTTTGCTTCATTTTCTAATTACTTTTACAGGGAAACTGTTTTTACGCGCCATGGAGCTTGTGACACTCTGTGATCTCTCAAAGCTTGCAATTCTCC |

**Supplementary Table 6. sRNA library adaptors and sRNA barcodes (sRBC, indicated as XX XXX)**

| **3’ adaptor** | **/5rApp/NN NNN NXX XXX AGA TCG GAA GAG CAC ACG TCT /3ddC/** |
| --- | --- |
| sRBC-1001 | CAGTG |
| sRBC-1002 | AGCAA |
| sRBC-1003 | GGTAT |
| sRBC-1004 | TACCA |
| sRBC-1005 | GTCAG |
| sRBC-1006 | TGACT |
| sRBC-1008 | CGTTC |
| sRBC-1009 | ATGGA |
| sRBC-1010 | GAACG |
| sRBC-1011 | ACGAG |
| 5’ adaptor | ACACUCUUUCCCUACACGACGCUCUUCCGAUCUNNNN |

**Supplementary Table 7. Sequencing data generated in this study**

| **Experiment** | **Description** | **Library ID** | **Data in** | **Method** |
| --- | --- | --- | --- | --- |
|  |  |  |  |  |
| RNA-seq of F1 from reciprocral crosses between NIC203 and EG6180 parental lines | Maternal inheritance of slow-1/grow-1 | INK531xEG6180_mRNA_1 (repeat 1-3) | Extended Data Fig. 1g | mRNA-seq |
|  | Maternal inheritance of slow-1/grow-1 | INK531xEG6180_mRNA_2 (repeat 4-6) | Extended Data Fig. 1g | mRNA-seq |
|  | Paternal inheritance of slow-1/grow-1 | QX2355xNIC203_mRNA_1  (repeat 1-3) | Extended Data Fig. 1g | mRNA-seq |
|  | Paternal inheritance of slow-1/grow-1 | QX2355xNIC203_mRNA_2  (repeat 4-6) | Extended Data Fig. 1g | mRNA-seq |
| RNA-seq of F1 from reciprocral crosses between NIL and EG6180 lines | Maternal inheritance of slow-1/grow-1 | INK255xEG6180_mRNA_1  (repeat 1-3) | Fig. 1c | mRNA-seq |
|  | Maternal inheritance of slow-1/grow-1 | INK255xEG6180_mRNA_2  (repeat 4-7) | Fig. 1c | mRNA-seq |
|  | Paternal inheritance of slow-1/grow-1 | QX2355xQX2345_mRNA_1  (repeat 1-3) | Fig. 1c | mRNA-seq |
|  | Paternal inheritance of slow-1/grow-1 | QX2355xQX2345_mRNA_2  (repeat 4-6) | Fig. 1c | mRNA-seq |
| Sequencing of *slow-1* promoter deletion strain | slow-1 promoter deletion strain in NIL grow-1.2 background | DELprom | Extended Data Fig. 7b | mRNA-seq |
|  | NIL grow-1.2 strain | INK249_repeat1 | Extended Data Fig. 7b | mRNA-seq |
|  | NIL grow-1.2 strain | INK249_repeat2 | Extended Data Fig. 7b | mRNA-seq |
|  | NIL grow-1.2 strain | INK249_repeat3 | Extended Data Fig. 7b | mRNA-seq |
| Assembly of EG6180 parental strain | Single molecule long-read sequencing of EG6180 | EG6180.nanopore | Extended Data Fig. 8a | WGS |
| EG6180 transcriptome for genome annotation | mRNA-seq of EG6180 (mixed developmental stages) | EG6180.mRNA.mixed | Extended Data Fig. 8a | mRNA-seq |
| NIL parental line | NIL WT line | QX2345_repeat1 | Extended Data Fig. 1c | mRNA-seq |
|  | NIL WT line | QX2345_repeat2 | Extended Data Fig. 1c | mRNA-seq |
|  | NIL WT line | QX2345_repeat3 | Extended Data Fig. 1c | mRNA-seq |
| EG6180 parental line | EG6180 parental strain | EG6180_repeat1 | Extended Data Fig. 1c | mRNA-seq |
|  | EG6180 parental strain | EG6180_repeat2 | Extended Data Fig. 1c | mRNA-seq |
|  | EG6180 parental strain | EG6180_repeat3 | Extended Data Fig. 1c | mRNA-seq |
| sRNA sequencing of EG6180, NIC203, *prg1.1* and *prg1.2* strains | sRNA of EG6180 parental strain | EG6180_sRNA | Fig. 3e, Extended Data Fig. 9e | small RNA sequencing |
|  | sRNA of NIC203 parental strain | NIC203_sRNA | Extended Data Fig. 9e | small RNA sequencing |
|  | sRNA of *prg1.1* KO | prg-1.1(-)_sRNA | Supplementary Data 2 | small RNA sequencing |
|  | sRNA of *prg1.2* KO | prg-1.2(-)_sRNA | Supplementary Data 2 | small RNA sequencing |
| sRNA sequencing of parental NIL and F4 following TA paternal inheritance | sRNA of NIL control | QX2345(NIL)_sRNA | Fig. 3i, j | small RNA sequencing |
|  | sRNA of F4 repressed TA | F4_INK112xINK460_sRNA | Fig. 3i, j, Extended Data Fig. 5g | small RNA sequencing |
| PRG-1.1 and PRG-1.2 RIP | PRG-1.1 RIP | 3xFLAG::prg1.1_IP_sRNA | Extended data Fig. 4b,c | RIP-Seq |
|  | PRG-1.2 RIP | 3xFLAG::prg1.2_IP_sRNA | Extended Data Fig. 4b,c | RIP-Seq |
| H3K9me3 ChIP-seq of NIL and F4 following TA paternal inheritance | NIL input | QX2345(NIL)_input | Fig. 3k | ChIP-Seq |
|  | F4 input | F4_INK112xINK460_input | Fig. 3k | ChIP-Seq |
|  | NIL H3K9me3 ChIP-seq | QX2345(NIL)_ChIPseq | Fig. 3k | ChIP-Seq |
|  | F4 H3K9me3 ChIP-seq | F4_INK112xINK460_ChIPseq | Fig. 3k | ChIP-Seq |

**Supplementary Discussion**

**The *slow-1/grow-1* TA has two redundant antidotes**

We previously reported the existence of a maternal-effect TA in NIC203 Chr. III and identified the genes encoding the toxin and its cognate antidote, *slow-1* and *grow-1*, using CRISPR/Cas9-aided HDR repair^9^. While generating new mutant lines for the present study, we noticed that a *grow-1* mutant line was phenotypically WT despite carrying an active toxin. This result suggested the presence of an additional antidote. Upon careful inspection of the NIC203 *de novo* genome assembly and the underlying Nanopore raw reads, we discovered that there were two copies of the *grow-1* antidote instead of one. These two copies, which we named *grow-1.1* and *grow-1.2*, were identical at the nucleotide level and in very close proximity, which likely caused an error in the genome assembler. In retrospect, we were fortunate while generating the original *grow-1* mutant in the *slow-1* null background. The *grow-1* gRNA we used targeted both copies and, unbeknownst to us, we had obtained a *grow-1.1* and *grow-1.2* double mutant. To facilitate our work with *grow-1.1* and *grow-1.2*, we designed gene specific primers pairs. Since these two genes are identical and redundant, we collectively refer to them as *grow-1* in this manuscript. In other words, all grow-1 mutants are *grow-1.1* *grow-1.2* double mutants. On the other hand, *grow-2* only has one copy in the genome.

**Difference between a maternal-effect TA and parent-of-origin effects**

TAs are made up of two linked genes: a toxin and its cognate antidote. Toxins are expressed in the germline of carriers, whereas antidotes are expressed zygotically. Except for the *C. elegans peel-1/zeel-1* TA, all other known TAs have a maternal-effect, that is, their toxic activity is transmitted through the maternal germline. This can be easily shown by performing reciprocal crosses between heterozygous TA carriers and homozygous non-carriers. In the case of maternal-effect TA, a cross between heterozygous mothers and homozygous males results in 50% affected progeny, whereas a cross between heterozygous males and homozygous mothers results in no affected progeny. It is also worth noting that in all maternal-effect toxins for which data is available, the toxins are loaded as protein into unfertilized eggs, analogously to maternal factors that are normally provisioned to ensure proper embryonic development. On the other hand, parent-of-origin effects occur when the phenotypic effects of a gene are determined by whether the gene is inherited from the mother or the father. In mammals, epigenetic imprinting is an example of a parent-of-origin effect. In contrast to all previously known TAs, the *slow-1/grow-1* TA has a parent-of-origin effect because it is only active when inherited through the maternal lineage. In summary, maternal and parent-of-origin effects describe two different phenomena. The first one is primarily concerned with the tissue where the toxin is expressed (either eggs or sperm), whereas the second one is concerned with differences in the activity or expression of the toxin between maternally and paternally inherited alleles.

**On epigenetic licensing and related terminology**

In this manuscript, we used the term “epigenetic licensing” as originally coined by Johnson and Spence (2011) when studying *C. elegans* *fem-1*. The authors found that maternal transcripts of the sex-determining gene *fem-1* are required to license expression of a wild-type *fem-1* allele in the zygotic germ line. Thus, “epigenetic licensing” requires establishing a causal link between the presence of maternal transcript and the activation of the cognate zygotic gene. In later studies, the term “licensing” has also been used to describe the role of the Argonaute CSR-1 in counteracting piRNA silencing. For instance, Seth and colleagues (2013) found that RNAa “licenses” previously silenced transgenes and Wedeles and colleagues (2013) found that tethering of CSR-1 was sufficient to “license” their expression of silenced transgenes. However, it is important to mention that neither of these two studies established a link between this “licensing” activity and maternal transcripts. Importantly, whether CSR-1 activity is required for *fem-1* epigenetic licensing has not been shown to date. Moreover, a recent study that identified CSR-1 “targets” on a genome wide-scale failed to identify *fem-1* among them^90^. Thus, it cannot be assumed that “epigenetic licensing” by maternal transcripts is equivalent to “licensing” by CSR-1.

**Supplementary Notes**

**Supplementary Note 1**

**PRG-1.1 and PRG-1.2 are redundant but non-equivalent**

To explore the redundancy between PRG-1.1 and PRG-1.2, we immunoprecipitated 3xFLAG::PRG-1.1 and 3xFLAG::PRG-1.2 from gravid hermaphrodites and sequenced their associated sRNAs in biological triplicates (Extended Data Fig. 4a,b). As expected, sRNA bound by these two proteins were highly enriched in piRNAs compared with total sRNA library across replicates—80.4% (70-85%) vs. 9.2% (7-11%), respectively. Most piRNAs—including *Ctr-*21ur-06949 and *Ctr-*21ur-06917—were bound by both PRG-1.1 and PRG-1.2, suggesting that their redundancy stems from targeting a common set of transcripts (Extended Data Fig. 4b, Supplementary Data 2). We also identified a subset of 787 (2.9%) piRNAs that exhibit differential binding between the two Argonautes after correcting for multiple testing (Supplementary Data 2). 72.6% of these piRNAs also showed differential downregulation in *prg-1.1* and *prg-1.2* mutants compared to WT, with a majority (87.9%) preferentially bound by PRG-1.1 (Supplementary Data 2). Notably, these differentially bound piRNAs were not randomly distributed across Chr. IV but they clustered in three distinct genomic regions (3,9-4,8Mb, 5,2-6,2Mb; and 9,5-10,5Mb, Extended Data Fig. 4c, Supplementary Data 2), which suggests shared genomic features among them and is consistent with the diversification of piRNAs via local duplication and modification of pre-existing sequences^31^. Our findings indicate that PRG-1.1 and PRG-1.2 have mostly overlapping, but not entirely equivalent, piRNA binding preferences, likely contributing to their differential impact on *slow-1* repression.

**Supplementary Note 2**

## **The *slow-1* toxin is not regulated by ‘RNAa’**

CSR-1 is unique among all worm Argonautes because it can activate silenced transgenes via ‘RNAa’ (RNA-induced epigenetic gene activation)^1^. Furthermore, tethering CSR-1 to transgenes can protect them from piRNA silencing^2^. *C. elegans* codes for two CSR-1 isoforms: a long one, CSR-1a, which is expressed in spermatogenic gonad and a shorter one, CSR-1b, which is constitutively expressed in the oogenic gonad^3,4^. We identified a single homolog of *csr-1* in the genome of *C. tropicalis.* Tagging of the endogenous *csr-1* with either an N-terminal or an internal FLAG epitope revealed that both long and short isoforms are also expressed in *C. tropicalis* and immunofluorescence of early embryos was consistent with their known expression pattern in *C. elegans* (Extended Data Fig. 6b,c).

To test whether CSR-1 is required for *slow-1* expression following maternal inheritance, we engineered a *C. tropicalis csr-1* null mutant disrupting both isoforms in the EG6180 background. A small fraction of *C. tropicalis* null mutants was partially fertile and homozygous *csr-1(-)* lines could be stably propagated for multiple generations despite extensive embryonic lethality in the population (Extended Data Fig. 6d). In agreement with the role of CSR-1 in *C. elegans*^5^, DAPI staining of *csr-1(-)* null embryos revealed chromosome segregation problems, such as anaphase bridging events (Extended Data Fig. 6e), suggesting that CSR-1 plays a similar role in both species. In *C. elegans*, ‘RNAa’ is abolished in the F_1_ progeny if either of the parents is a heterozygous carrier of a null *csr-1* allele^1^. Thus, to test whether maternal *csr-1* is necessary for the parent-of-origin effect, we crossed *csr-1(-); slow-1/grow-1* hermaphrodites to EG6180 males (see methods) and inspected their F_2_ progeny (Extended Data Fig. 6f). We found that 96.8% (n=32) of EG/EG F_2_ individuals were developmentally delayed, thus indicating that expression of maternally inherited *slow-1* was not impaired in the absence of maternal CSR-1. Unfortunately, although a small fraction of *C. tropicalis* *csr-1(-)* hermaphrodites was viable and fertile, the high incidence of abnormal phenotypes in these worms precluded us from confidently testing the combined effect of maternal and zygotic *csr-1* loss of function. However, our results strongly suggest that CSR-1 is not behind the parent-of-origin effect^6^.

**Supplementary Note 3**

## **A related *slow-2/grow-2* TA has no parent-of-origin effect**

The presence of the *slow-1/grow-1* TA in the NIL explains why F_2_ non-carrier progeny (EG/EG) are delayed (Fig. 1a). However, it does not account for the fact that ~1/3 of F_2_ homozygous TA carriers (NIC/NIC) are also delayed in crosses between the NIL and EG6180 (Fig. 1b). We previously hypothesized that this inheritance pattern could emerge from an independent incompatibility factor present in EG6180 within the homologous boundaries of the NIC203 introgression^7^. To investigate this, we sequenced and assembled the EG6180 genome using a combination of Nanopore long-reads and Illumina short-reads and annotated the genome using RNA-seq data (see methods). We then searched for genes that were either missing, mutated or highly divergent within the NIC203 40kb introgression region but present in EG6180. We found a pair of highly divergent but homologous genes to the *slow-1/grow-1* TA and named the new element *slow-2/grow-2* (Extended Data Fig. 8a). *slow-2* transcripts were readily detectable in EG6180 but absent in the NIL (Fig. 5b and Extended Data Fig. 9a).

To test whether the *slow-2/grow-2* locus is a *bona fide* TA, we generated a *slow-2(-)*/*grow-2(-)* double mutant strain using CRISPR/Cas9 in the parental EG6180 background. Both mutant alleles introduce a premature stop codon that is predicted to generate a null allele (Extended Data Fig. 9b,c). We then crossed *slow-2(-)/grow-2(-)* double mutant hermaphrodites to WT EG6180 males and inspected their F_2_ progeny (Fig. 5c). If these two genes encode a toxin and an antidote, we would expect the double mutant to behave like a NIC203 susceptible allele^7^. Indeed, we observed 23.1% delayed worms among the F_2_ progeny (n=160), while 87% (n=31) of *slow-2(-)/grow-2(-)* double mutant individuals were developmentally delayed (Extended Data Fig. 8), in agreement with the TA model. As a control, we observed only background levels of developmental defects in the *slow-2(-)* single mutant and *slow-2(-)/grow-2(-)* double mutant parental lines (3.8%; n=130 and 0.72%; n=139, respectively).

Despite having a common evolutionary origin, *slow-1/grow-1* and *slow-2/grow-2* are highly divergent. SLOW-1 and SLOW-2 are only 32.6% identical at the protein level (Extended Data Fig. 9d). In crosses between the NIL and EG6180, each TA selectively targets individuals homozygous for the other element (Fig. 1b, 5d). As a result, both TAs are in direct genomic conflict, and their drive activity is predicted to be severely hampered^7^. However, there is one key difference between the two: *slow-2/grow-2* does not show a parent-of-origin effect (Extended Data Fig. 8d). In agreement with this, we found no difference in *slow-2* expression levels among F_1_ in NIL and EG6180 reciprocal crosses (Extended Data Fig. 8e). 22G-RNAs derived from *slow-1* were 10 times more abundant than those derived from *slow-2* (Extended Data Fig. 9e; p=0.042) while no global differences in 22G-RNA abundance were observed between NIC203 and EG6180 (Extended Data Fig. 8e). We speculate that the high levels of genetic divergence observed between *slow-1* and *slow-2* could be explained in part by an evolutionary arms race between the two TAs originating from their antagonistic action and involving continues cycles of positive selection and counter-adaptation. However, a formal test of this model would require population genetic modeling and allele frequency measurements derived from wild populations.

**Supplementary References**

1. Seth, M. *et al.* The C. elegans CSR-1 Argonaute Pathway Counteracts Epigenetic Silencing to Promote Germline Gene Expression. *Developmental Cell* **27**, 656–663 (2013).

2. Wedeles, C. J., Wu, M. Z. & Claycomb, J. M. Protection of Germline Gene Expression by the C. elegans Argonaute CSR-1. *Developmental Cell* **27**, 664–671 (2013).

3. Charlesworth, A. G. *et al.* Two isoforms of the essential C. elegans Argonaute CSR-1 differentially regulate sperm and oocyte fertility. *Nucleic Acids Research* **49**, 8836–8865 (2021).

4. Nguyen, D. A. H. & Phillips, C. M. Arginine methylation promotes siRNA-binding specificity for a spermatogenesis-specific isoform of the Argonaute protein CSR-1. *Nat Commun* **12**, 4212 (2021).

5. Claycomb, J. M. *et al.* The Argonaute CSR-1 and Its 22G-RNA Cofactors Are Required for Holocentric Chromosome Segregation. *Cell* **139**, 123–134 (2009).

6. Gerson-Gurwitz, A. *et al.* A Small RNA-Catalytic Argonaute Pathway Tunes Germline Transcript Levels to Ensure Embryonic Divisions. *Cell* **165**, 396–409 (2016).

7. Ben-David, E. *et al.* Ubiquitous Selfish Toxin-Antidote Elements in Caenorhabditis Species. *Current Biology* **31**, 990-1001.e5 (2021).
