# Supplementary material for: Selfish conflict underlies RNA-mediated parent-of-origin effects
Source: Nature. 2024 Mar 6;628(8006):122–9. doi: 10.1038/s41586-024-07155-z (PMC10990930; doi:10.1038/s41586-024-07155-z)
Supplement: Supplementary file 2 — Reporting Summary [file 41586_2024_7155_MOESM2_ESM.pdf]

nature portfolio

Corresponding author(s): Alejandro Burga

Last updated by author(s): 2023/11/09

Reporting Summary

Nature Portfolio wishes to improve the reproducibility of the work that we publish. This form provides structure for consistency and transparency in reporting. For further information on Nature Portfolio policies, see our Editorial Policies and the Editorial Policy Checklist.

Statistics

For all statistical analyses, confirm that the following items are present in the figure legend, table legend, main text, or Methods section.

n/a	Confirmed	
☐	☒	The exact sample size (*n*) for each experimental group/condition, given as a discrete number and unit of measurement
☐	☒	A statement on whether measurements were taken from distinct samples or whether the same sample was measured repeatedly
☐	☒	The statistical test(s) used AND whether they are one- or two-sided<br>*Only common tests should be described solely by name; describe more complex techniques in the Methods section.*
☒	☐	A description of all covariates tested
☒	☐	A description of any assumptions or corrections, such as tests of normality and adjustment for multiple comparisons
☐	☒	A full description of the statistical parameters including central tendency (e.g. means) or other basic estimates (e.g. regression coefficient) AND variation (e.g. standard deviation) or associated estimates of uncertainty (e.g. confidence intervals)
☐	☒	For null hypothesis testing, the test statistic (e.g. *F*, *t*, *r*) with confidence intervals, effect sizes, degrees of freedom and *P* value noted<br>*Give P values as exact values whenever suitable.*
☒	☐	For Bayesian analysis, information on the choice of priors and Markov chain Monte Carlo settings
☒	☐	For hierarchical and complex designs, identification of the appropriate level for tests and full reporting of outcomes
☒	☐	Estimates of effect sizes (e.g. Cohen's *d*, Pearson's *r*), indicating how they were calculated

Our web collection on statistics for biologists contains articles on many of the points above.

Software and code

Policy information about availability of computer code

Data collection	N/A
Data analysis	Flye Assembler v2.7.1 , Sibelia, Ragout, DEseq2, STAR, Cutadapt, samtools v1.18, hisat2 v2.1, seqkit v0.13 , Gviz, ViennaRNA Package v2.0.58, blast v2.2.26, Fiji (v1.53r)

For manuscripts utilizing custom algorithms or software that are central to the research but not yet described in published literature, software must be made available to editors and reviewers. We strongly encourage code deposition in a community repository (e.g. GitHub). See the Nature Portfolio guidelines for submitting code & software for further information.

Data

Policy information about availability of data

All manuscripts must include a data availability statement. This statement should provide the following information, where applicable:

- Accession codes, unique identifiers, or web links for publicly available datasets
- A description of any restrictions on data availability
- For clinical datasets or third party data, please ensure that the statement adheres to our policy

Sequencing data are available under NCBI project PRJNA850171. Raw data for all genetic crosses including references to figures is found in Extended Data Table 1

Human research participants

Policy information about studies involving human research participants and Sex and Gender in Research.

Reporting on sex and gender	It does not apply to our study
Population characteristics	It does not apply to our study
Recruitment	It does not apply to our study
Ethics oversight	It does not apply to our study

Note that full information on the approval of the study protocol must also be provided in the manuscript.

Field-specific reporting

Please select the one below that is the best fit for your research. If you are not sure, read the appropriate sections before making your selection.

☒ Life sciences ☐ Behavioural & social sciences ☐ Ecological, evolutionary & environmental sciences

For a reference copy of the document with all sections, see nature.com/documents/nr-reporting-summary-flat.pdf

Life sciences study design

All studies must disclose on these points even when the disclosure is negative.

Sample size	For each genetic cross, we phenotyped at least 100 F2 individuals. This is in our experience sufficient to significantly distinguish between an active or inactive toxin-antidote element (segreation of a single locus Mendelian trait).
Data exclusions	We excluded from the analysis the progeny of individuals resulting from self-fertilization (genotyping of F1 by PCR). Progeny from heterozygous F1 individuals that could not be genotyped due to to technical problems were noted as "n.g." and included in the analyses.
Replication	All genetic crosses were independently performed at least twice starting from independent nematode cultures. And each replicate included ten independent F1 individuals and 10 F2 progeny per F1 (on average). All raw numbers can be found on Extended Data Table 1
Randomization	We did not perform randomization
Blinding	Phenotypic scoring of all F2 progeny was performed prior and independently of genotyping.

Reporting for specific materials, systems and methods

We require information from authors about some types of materials, experimental systems and methods used in many studies. Here, indicate whether each material, system or method listed is relevant to your study. If you are not sure if a list item applies to your research, read the appropriate section before selecting a response.

Materials & experimental systems

n/a	Involved in the study	
☐	☒	Antibodies
☒	☐	Eukaryotic cell lines
☒	☐	Palaeontology and archaeology
☐	☒	Animals and other organisms
☒	☐	Clinical data
☒	☐	Dual use research of concern

Methods

n/a	Involved in the study	
☐	☒	ChIP-seq
☒	☐	Flow cytometry
☒	☐	MRI-based neuroimaging

Antibodies

Antibodies used	Membranes were incubated with anti-FLAG M2 (mouse,1:2000, Sigma-Aldrich, F3165) or anti-Actin (rabbit, 1:3000, Abcam, ab13772) primary antibody in a blocking solution overnight at 4°C.
Validation	Both antibodies are highly used commercial antibodies. We have also validated the anti-FLAG M2 antibody for WB and immunofluoresence in C. tropicalis using negative controls (lines without FLAG tag)

Animals and other research organisms

Policy information about studies involving animals; ARRIVE guidelines recommended for reporting animal research, and Sex and Gender in Research

Laboratory animals	In this study we only used previously characterized C. tropicalis nematode lines NIC203 and EG6180 described in Ben-David et al. Current Biology (2021) All mutants lines are available in Extended Table 3
Wild animals	N/A
Reporting on sex	N/A
Field-collected samples	N/A
Ethics oversight	N/A

Note that full information on the approval of the study protocol must also be provided in the manuscript.

ChIP-seq

Data deposition

☒ Confirm that both raw and final processed data have been deposited in a public database such as GEO.

☐ Confirm that you have deposited or provided access to graph files (e.g. BED files) for the called peaks.

Data access links *May remain private before publication.*	Sequencing data are available under NCBI project PRJNA850171.
Files in database submission	H3K9me3 ChIP and Input controls samples for two conditions (NIL and F4 population) are provided
Genome browser session (e.g. UCSC)	*Provide a link to an anonymized genome browser session for "Initial submission" and "Revised version" documents only, to enable peer review. Write "no longer applicable" for "Final submission" documents.*

Methodology

Replicates	three biological replicates
Sequencing depth	We aim to generate at least 20M reads per sample, which corresponds to ~25X coverage of the whole genome of C.tropcalis.
Antibodies	2 µg of anti-H3K9me3 antibody (Ab8898, Abcam)
Peak calling parameters	. Peaks were called by macs2 v2.2.5 with --broad and --mfold 1 50 options85
Data quality	*Describe the methods used to ensure data quality in full detail, including how many peaks are at FDR 5% and above 5-fold enrichment.*
Software	MACS2
